# Modeling Hypoxic Stress In Vitro Using Human Embryonic Stem Cells Derived Cardiomyocytes Matured by FGF4 and Ascorbic Acid Treatment

**DOI:** 10.3390/cells10102741

**Published:** 2021-10-14

**Authors:** Seung-Cheol Choi, Ha-Rim Seo, Long-Hui Cui, Myeong-Hwa Song, Ji-Min Noh, Kyung-Seob Kim, Ji-Hyun Choi, Jong-Ho Kim, Chi-Yeon Park, Hyung Joon Joo, Soon Jun Hong, Tae Hee Ko, Jong-Il Choi, Hyo Jin Kim, Jong-Hoon Kim, Se-Hwan Paek, Ji-Na Park, Dong-Hyung Kim, Yongjun Jang, Yongdoo Park, Do-Sun Lim

**Affiliations:** 1 Department of Cardiology, Cardiovascular Center, Korea University College of Medicine, 145 Anam-ro, Seongbuk-gu, Seoul 02841, Korea; choisc86@gmail.com (S.-C.C.); shl5764@gmail.com (H.-R.S.); longhuicui@gmail.com (L.-H.C.); songmh616@gmail.com (M.-H.S.); wlals5344@gmail.com (J.-M.N.); ssks30101@gmail.com (K.-S.K.); j2610@hanmail.net (J.-H.C.); mecey@naver.com (J.-H.K.); chiyeon19@gmail.com (C.-Y.P.); drjoohj@gmail.com (H.J.J.); psyche94@gmail.com (S.J.H.); 2 R&D Center for Companion Diagnostic, SOL Bio Corporation, Suite 510, 27, Seongsui-ro7-gil, Seongdong-gu, Seoul 04780, Korea; shpaek@sol-bio.com; 3 Division of Drug Efficacy Evaluation, New Drug Development Center, Osong Medical Innovation Foundation, 123, Osongsaengmyeong-ro, Osong-eup, Heungdeok-gu, Cheonju-si 28160, Korea; 4 Division of Cardiology, Department of Internal Medicine, Korea University Anam Hospital, Korea University College of Medicine, Seoul 02841, Korea; kotiti2000@hanmail.net (T.H.K.); jongilchoi@korea.ac.kr (J.-I.C.); 5 Laboratory of Stem Cells and Tissue Regeneration, Department of Biotechnology, College of Life Sciences and Biotechnology, Korea University, Seoul 02841, Korea; 010hyojin@naver.com (H.J.K.); jhkim@korea.ac.kr (J.-H.K.); 6 Abbott Diagnostics Korea Inc., 65 Borahagal-ro, Yongin 17099, Korea; jina.park@abbott.com; 7 Korea Research Institute of Standards and Science, 267 Gajeong-ro, Yuseong-gu, Deajeon 34113, Korea; donghyung.kim@kriss.re.kr; 8 Department of Biomedical Sciences, College of Medicine, Korea University, 145 Anam-ro, Seongbuk-gu, Seoul 02841, Korea; jyj727@korea.ac.kr (Y.J.); ydpark67@korea.ac.kr (Y.P.)

**Keywords:** pluripotent stem cells, cytokines, cardiac, differentiation, hypoxia

## Abstract

Mature cardiomyocytes (CMs) obtained from human pluripotent stem cells (hPSCs) have been required for more accurate in vitro modeling of adult-onset cardiac disease and drug discovery. Here, we found that FGF4 and ascorbic acid (AA) induce differentiation of BG01 human embryonic stem cell–cardiogenic mesoderm cells (hESC-CMCs) into mature and ventricular CMs. Co-treatment of BG01 hESC-CMCs with FGF4+AA synergistically induced differentiation into mature and ventricular CMs. FGF4+AA-treated BG01 hESC-CMs robustly released acute myocardial infarction (AMI) biomarkers (cTnI, CK-MB, and myoglobin) into culture medium in response to hypoxic injury. Hypoxia-responsive genes and potential cardiac biomarkers proved in the diagnosis and prognosis of coronary artery diseases were induced in FGF4+AA-treated BG01 hESC-CMs in response to hypoxia based on transcriptome analyses. This study demonstrates that it is feasible to model hypoxic stress in vitro using hESC-CMs matured by soluble factors.

## 1. Introduction

Heart disease and drug developmental studies have benefited from human pluripotent stem cell-derived cardiomyocytes (hPSC-CMs). However, hPSC-CMs resemble immature embryonic or fetal CMs rather than mature adult CMs, and therefore have limitations in disease modeling and pharmacological studies. Furthermore, hPSC-CMs are a mixture of CMs with atrial-, ventricular-, and nodal-like phenotypes. Therefore, generation of mature and subtype CMs from hPSCs is important for in vitro modeling of adult-onset cardiac disease and drug discovery. However, despite exciting progress, no strategy to date has yielded both functionally and structurally mature CMs [1].

A small number of chemical cues or soluble factors able to induce differentiation of hPSCs into mature ventricular CMs have been reported [1]. Thyroid hormone and glucocorticoids induce the maturation of different PSCs into immature CMs [2,3,4,5,6]. Ascorbic acid (AA) enhances the cardiac differentiation of PSCs by promoting the proliferation of cardiac progenitor cells [7]. In addition, small molecules (Wnt signaling inhibitor, Rho kinase inhibitor) were shown to induce mature ventricular cardiac differentiation of PSCs [8,9]. 

Fibroblast growth factors (FGFs), which function as paracrine growth factors or endocrine hormones, have diverse functions in development, health, and disease [10]. FGF2 controls the differentiation of resident cardiac precursors into functional CMs in mice [11]. FGF2 and FGF4, when ectopically expressed in chick embryos, can initiate cardiac development by inducing host tissues [12]. FGF2 and FGF10 have been shown to stimulate the cardiac differentiation of cultured stem cells and cardiac reprogramming of cultured fibroblasts [10]. FGF8, FGF9, FGF10, and FGF16 act as paracrine signals in embryonic heart development, and in postnatal heart pathophysiology [10]. FGF signaling was shown to enforce cardiac chamber identity in the developing ventricle in zebrafish [13]. These previous studies suggest that FGF signaling could regulate CM differentiation and maturation in a FGF member-specific manner.

Cardiac Troponin I (cTnI) shows both high specificity and high diagnostic accuracy for acute myocardial infarction (AMI) and remains in the bloodstream for a prolonged time compared to other cardiac biomarkers [14,15,16]. Creatine kinase MB (CK-MB) rises in the serum 4–9 h after myocardial injury, and returns to baseline at 48–72 h [17]. Although cTnI and CK-MB are cardiac-specific markers, they appear in the peripheral blood stream 3–4 h after onset [18]. Myoglobin is an early indicator of AMI because it appears 1–3 h after onset. However, myoglobin is non-specific [19,20]. Thus, multiple cardiac markers are usually employed at the same time for early and specific detection of AMI onset [20,21,22]. 

In this study, we screened various chemicals, cytokines, and growth factors under a feeder-free culture system and found that FGF4 and AA induced differentiation of BG01 human embryonic stem cell-cardiogenic mesoderm cells (hESC-CMCs) into mature ventricular CMs at the expense of nodal CMs. We investigated the feasibility of using FGF4+AA-treated BG01 hESC-CMs as an in vitro hypoxic stress model by evaluating the release of AMI-specific biomarkers (cTnI, CK-MB, and myoglobin) into the media by normoxia or hypoxia-treated hESC-CMs cultured without or with FGF4+AA. Finally, we investigated whether FGF4+AA-treated BG01 hESC-CMs are suitable for in vitro hypoxic stress modeling at the molecular level by analyzing the transcriptomes of FGF4+AA-treated BG01 hESC-CMs cultured under normoxic or hypoxic environments for 24 h. 

## 2. Materials and Methods

### 2.1. Culture and Cardiac Differentiation of hESCs

A hESC line, BG01 was obtained from the WiCell Research Institute (Madison, WI, USA). BG01 hESCs were maintained on Matrigel-coated plates in E8 medium (Thermo Fisher Scientific, Waltham, MA, USA). For cardiac differentiation, BG01 hESCs were dissociated into single cells with Accutase (Sigma-Aldrich, Saint Louis, MO, USA) and then seeded onto a Matrigel-coated at 32,000 cells/cm^2^ in E8 supplemented with 2 μM Thiazovivin (Sigma-Aldrich), a Rho kinase inhibitor from day −3 to day 0. At day 0, cells were treated with 6 µM CHIR99021 (Sigma-Aldrich) in the basal medium RPMI 1640 (Thermo Fisher Scientific), L-Ascorbic acid 2-phosphate sesquimagnesium salt hydrate (Sigma-Aldrich), and bovine serum albumin (BSA, Sigma-Aldrich) medium for 1 day. At 2 days, cells were treated with 2 µM IWP2 (Tocris Bioscience, Ellisville, MO, USA) in RPMI 1640 + B27 minus insulin (RPMI/B27-Insulin) medium for 48 h. Cells were treated with 10 ng/mL FGF2 (Peprotech, Rocky Hill, NJ, USA), 5, 10, 25, 50, and 100 ng/mL FGF4 (Sigma-Aldrich), 10 ng/mL FGF10 (Peprotech), 200 µg/mL AA, or 10 ng/mL FGF4 + 200 µg/mL AA (FGF4+AA) in RPMI/B27-Insulin medium from day 5 to day 15. 

### 2.2. Quantitative Reverse Transcription Polymerase Chain Reaction (qRT-PCR)

Total RNA was extracted from cells with TRIzol (MRC Inc. Cincinnati, OH, USA) according to the manufacturer’s protocol. The concentration of total RNA was measured using a NanoDrop spectrophotometer (Thermo Fisher Scientific), and 500 ng of total RNA was used for complementary DNA synthesis by supplementation with M-MLV reverse transcriptase (Invitrogen, Seoul, Korea) at 37 °C for 50 min in a 20 μL volume. qRT-PCR was performed using SYBR Green Mixture (Bio-Rad Laboratories, Hercules, CA, USA), and the results were recorded using an MYiQ2 Detection System (Bio-Rad Laboratories, Hercules, CA, USA). The relative gene expression levels were quantified based on Ct and normalized to the reference gene, *GAPDH* or *β-ACTIN*. To avoid genomic DNA amplification, intron spanning primers were designed using the ProbeFinder software (https://lifescience.roche.com/global_en/articles/Universal-ProbeLibrary-System-Assay-Design.html/, accessed on 10 October 2021). Primer sequences are listed in Table S1.

### 2.3. Immunofluorescence Staining

CMs were washed twice with phosphate-buffered saline (PBS) and fixed with 4% paraformaldehyde dissolved in PBS for 20 min. The fixed cells were permeabilized with 0.1% Triton X-100 in PBS for 30 min, washed in PBS + 0.1% Tween 20 (PBST), and blocked with 5% normal goat serum (NGS, Thermo Fisher Scientific) in PBST. The cells were stained with the following primary antibodies: anti-cTnT (Thermo Fisher Scientific), anti-α-actinin (Sigma-Aldrich), anti-MLC2v (Proteintech, Rosemont, IL, USA), anti-MLC2a (Synaptic Systems, Goettingen, Germany), and anti-cleaved caspase 3 (Cell Signaling Technology, Danvers, MA, USA) antibodies at 4 °C overnight in 2% NGS in PBST. The cells were washed twice in PBST and incubated for 1 h with the following secondary antibodies: Alexa Fluor 488 anti-mouse IgG1, Alexa Fluor 594 goat anti-mouse IgG2b, and Alexa Fluor 594 goat anti-rabbit IgG (all from Molecular Probes, Eugene, OR, USA). The nuclei were stained with DAPI, and the stained cells were mounted using a fluorescent mounting solution (DAKO, Carpinteria, CA, USA). Immunofluorescence images were acquired using a fluorescence microscope (Olympus-Europa GmbH, Hamburg, Germany) and a confocal fluorescence microscope (Carl Zeiss, Oberkochen, Germany).

### 2.4. Beating Analysis Using Captured Movies

Contractile properties of BG01 hESC-CMs were estimated by analyzing the movies taken with a microscope (Nikon, Tokyo, Japan) at 50× magnification. Movies were captured at 50 frames per second and then analyzed by NIS software (Nikon), which counts the variation in light intensity in a selected region during 10 s. The beating kinetics of each sample were estimated to identify beating regularity and homogeneity.

### 2.5. AMI Biomarker Analysis Using In Vitro Hypoxic Model

For hypoxic experiment, BG01 hESCs were treated without or with FGF4+AA in RPMI/B27-Insulin medium from day 5 to day 15. After washing with RPMI/B27-Insulin medium, BG01 hESC-CMs in the hypoxia group were cultured in a humidified incubator at 37 °C with a gaseous mixture of 2% O_2_, 94% N_2_, and 5% CO_2_ from day 15 to day 21. BG01 hESC-CMs in the normal group were cultured in the same condition except the O_2_ concentration was 21%. Cell media were collected on days 11, 13, 15, 17, 19, and 21 of differentiation. Collected media were centrifuged at 12,000× *g* for 5 min. Samples were kept frozen at −80 °C until analysis. A 1:1 mixture of 50 µL of cell medium with 50 µL of PBS + 5% BSA was prepared. AMI biomarker measurements were performed using myoglobin (LSI Medience Corp., Tokyo, Japan), CK-MB (LSI Medience Corp.), and hs-cTnI reagent (LSI Medience Corp.), on the PATHFAST cardiac biomarker analyzer (LSI Medience Corp.), in accordance with the manufacturer’s instructions.

### 2.6. RNA Sequencing (RNA-Seq) Analyses

Total RNA was extracted from normoxia (21% O_2_)- and hypoxia (2% O_2_)-treated BG01 hESC-CMs for 24 h after treatment with FGF4+AA between days 5 and 15 of differentiation with TRIzol reagent. The concentration of total RNA was measured using a NanoDrop spectrophotometer, RNA quality was assessed using the Agilent 2100 bioanalyzer with the RNA 6000 Nano Chip (Agilent Technologies, Amstelveen, The Netherlands). For each RNA sample, the library construction was performed using QuantSeq Library Prep kit (Lexogen, Inc., Vienna, Austria) according to the manufacturer’s instructions. High-throughput sequencing was performed using NextSeq. 500 (Illumina, Inc., San Diego, CA, USA). The whole transcriptomes were compared, and we identified differentially expressed genes that displayed a greater than 2-fold change in expression under hypoxic condition for 24 h. Differentially expressed genes were analyzed using the ExDEGA software (EBIOGEN, Inc., Seoul, Korea). Gene classification was based on searches performed using the DAVID (http://david.ncifcrf.gov/, accessed on 10 October 2021), Medline databases (http://www.ncbi.nlm.nih.gov/, accessed on 10 October 2021), KEGG pathway (http://www.genome.jp/kegg/mapper.html/, accessed on 12 October 2021) and STRING (http://www.string-db.org/, accessed on 10 October 2021). Heat maps of hypoxia-induced genes in hESC-CMs were performed using software MultiExperiment Viewer (Mev) [23]. Raw RNA-sequencing data have been deposited in the Gene Expression Omnibus (accession number GEO: GSE171824).

### 2.7. Statistical Analysis

All statistical values are expressed as the mean ± standard deviation (SD). Significant differences between means were determined by the Student’s *t*-test or analysis of variance (ANOVA) followed by the Student–Newman–Keuls test. Statistical significance was set at *p* <  0.05. All statistical analyses were performed using SigmaStat3.5 (SPSS, Chicago, IL, USA).

## 3. Results

### 3.1. FGF4 and AA Induce Differentiation of BG01 hESC-CMCs into Mature Ventricular CMs

To find factors that induce cardiac maturation and subtype differentiation of BG01 hESC-CMCs, we developed a cardiac differentiation protocol with Wnt modulation in modified chemically defined medium consisting of three components (mCDM3) consisting of RPMI 1640 basal medium, AA, and BSA. To determine whether cardiogenic mesoderm differentiation was induced, we examined the gene expression patterns of cell lineage markers in samples collected from days 1–5 of differentiation. Pluripotent markers (POU5F1, NANOG) were rapidly downregulated at day 2 with concurrent upregulation of mesendoderm markers (T, MIXL1) followed by upregulation of cardiogenic mesoderm markers (MESP1, VEGFR2) at day 5 (Figure S1). 

Among the selected factors that induce cardiac maturation and subtype differentiation, we further investigated the effects of FGF2, FGF4, FGF10, and AA on morphological changes and contraction properties after adding these factors at day 5 of differentiation for an additional 10 days (Figure S2A,B). Beating BG01 hESC-CMs were observed at approximately day 9 in untreated, FGF2-, FGF4-, FGF10-, and AA-treated groups (Figure S3). Different morphological changes and contraction properties were observed in a cytokine-specific manner on days 11–15 (Figure S3; Videos S1–S5). On approximately day 12, beating CMs begun to aggregate, and thick clump-like structures were present on day 15 in the FGF4-treated groups compared to the control group (Figure S3; Video S3). AA-treated hESC-CMs had a relatively flat morphology compared to the control group (Figure S2B; Video S5). However, morphological changes and beating contractions of hESC-CMs after FGF2- or FGF10-treatment were similar to those of the untreated control group (Figure S2B; Videos S2 and S4).

Next, we examined the effects of FGF2, FGF4, FGF10, and AA on expression levels of markers of CM maturation, subtypes, and cell lineages by qRT-PCR (Figure S2C). Levels of CD31, an endothelial cell marker were markedly increased in FGF2-treated hESC-CMs compared to untreated hESC-CMs, as were those of ANP, an atrial marker (Figure S2C). By contrast, expression of nodal CM markers (TBX18, HCN4) and smooth muscle cell markers (SMA, SM22) was decreased by FGF2 treatment (Figure S2C). FGF4 increased expression of a total CM marker (cTnT), a mature CM marker (cTnI), a ventricular CM marker (MLC2v), and atrial CM markers (MLC2a, ANP), whereas it decreased expression of nodal CM markers (TBX18, HCN4) and smooth muscle cell markers (SMA, SM22) (Figure S2C). Similarly, AA increased expression of a ventricular CM marker (MLC2v) and atrial CM markers (MLC2a, ANP), whereas it decreased expression of a nodal CM marker (TBX18) and smooth muscle cell markers (SMA, SM22) (Figure S2C). FGF10 slightly increased expression of ANP, TBX18, HCN4, and SM22 (Figure S2C). These results suggest that maturation and subtype specification of CMs as well as differentiation of smooth muscle and endothelial cells occurs in a cytokine-specific manner. We selected FGF4 and AA for further study because FGF4 and AA induced differentiation of BG01 hESC-CMCs into mature, and ventricular CMs.

FGF4 induced differentiation of hESC-CMCs toward ventricular and atrial CMs in the concentration range of 5 to 50 ng/mL (Figure S4). FGF4 concentrations between 10–25 ng/mL were more effective at inducing differentiation of hESC-CMCs into ventricular and atrial CMs (Figure S4). By contrast, FGF4 inhibited expression of TBX18, a nodal CM marker, in the concentration range of 5 to 100 ng/mL (Figure S4). Interestingly, significant reduction of SMA, a smooth muscle cell marker was observed in the FGF4 concentration range of 5 to 25 ng/mL (Figure S4).

### 3.2. Co-Treatment of FGF4+AA Synergistically Induces Differentiation of Immature BG01 hESC-CMCs into Mature Ventricular CMs 

**Figure 1:**
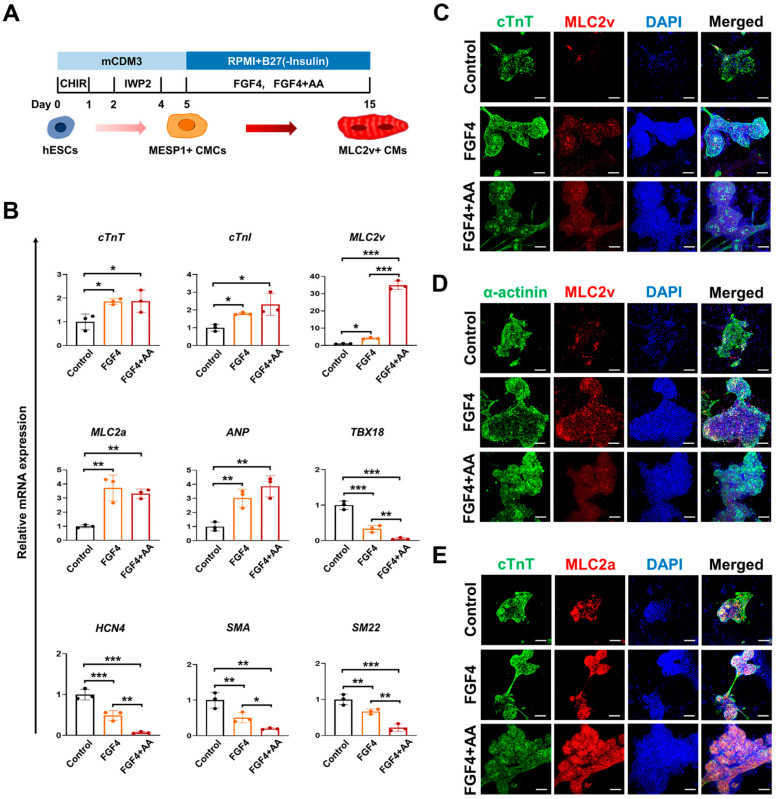
Co-treatment of FGF4+AA enhances differentiation of BG01 hESC-CMCs toward ventricular-like CMs but reduces differentiation into nodal-like CMs. (**A**) Schematic diagram of CM subtype differentiation protocol from BG01 hESC along with Wnt modulation followed by 10 ng/mL FGF4 or 10 ng/mL FGF4 + 200 µg/mL AA between days 5 and 15 of differentiation in mCDM3 and RPMI/B27(-Insulin) media. CHIR: CHIR99021; CM: cardiomyocyte; CMC: cardiogenic mesoderm cell; mCDM3: modified chemically defined medium consisting of three components. (**B**) qRT-PCR analysis of a total CM marker (cTnT), a mature CM marker (cTnI), a ventricular CM marker (MLC2v), atrial CM markers (MLC2a, ANP), nodal CM markers (TBX18, HCN4), and smooth muscle cell markers (SMA, SM22) in BG01 hESC-CMCs treated with 10 ng/mL FGF4 or 10 ng/mL FGF4 + 200 µg/mL AA in mCDM3 and RPMI/B27(-Insulin) media at differentiation day 15. Data were normalized to GAPDH level and expressed as relative values. Values represent means ± SDs. n = 3 for each group. * *p* < 0.05, ** *p* < 0.01 and *** *p* < 0.001 versus controls. Significant differences between the means of untreated-, FGF4-, and FGF4+AA-treated groups were analyzed by a one-way ANOVA followed by the Student–Newman–Keuls test. (**C**–**E**) Immunofluorescence images of BG01 hESC-CMs at differentiation day 15 after treatment with 10 ng/mL FGF4 or 10 ng/mL FGF4 + 200 µg/mL AA. hESC-CMs were stained with the indicated antibodies. Nuclei were stained with DAPI (blue). Scale bars = 100 μm.

We then examined if the combination of FGF4 and AA had a synergistic effect on the maturation and ventricular specification of hESC-CMCs because FGF4 or AA elevated transcript levels of mature and ventricular CM markers (Figure 1A). Interestingly, co-treatment of cells with FGF4+AA synergistically increased the expression of the ventricular CM marker, MLC2v, compared to treatment of cells with FGF4 alone (Figure 1B). FGF4+AA upregulated expression of MLC2v by as much as 35-fold (Figure 1B). By contrast, FGF4+AA strongly inhibited expression of nodal CM markers (TBX18, HCN4) and smooth muscle cell markers (SMA, SM22) compared to FGF4 or AA treatment alone during CM differentiation (Figure 1B).

We immunostained FGF4- or FG4+AA-treated hESC-CMs for a CM marker (cTnT), ventricular (MLC2v) and atrial (MLC2a) isoforms of myosin light chain, and α-actinin, a marker of sarcomeric organization, during cardiac differentiation. FGF4- or FG4+AA-treated hESC-CMs co-stained for cTnT and MLC2v, whereas untreated hESC-CMs were negative or weakly positive for MLC2v (Figure 1C). FGF4- or FG4+AA-treated CMs were α-actinin+/MLC2v+, whereas untreated control CMs were α-actinin+/MLC2v- (Figure 1D). Most cTnT+ CMs were also positive for MLC2a in untreated control, FGF4- or FG4+AA-treated CMs (Figure 1E).

**Figure 2:**
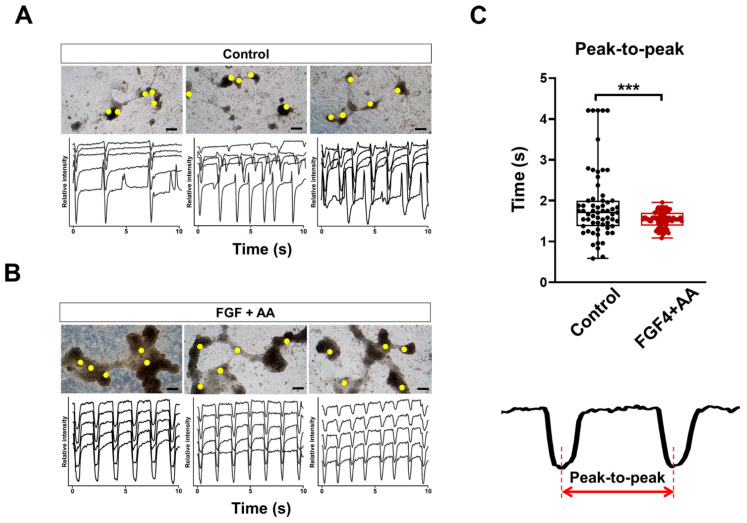
Co-treatment of FGF4+AA synchronize contraction of BG01 hESC-CMs. Beating characteristics at day 15 in (**A**) untreated CMs and (**B**) 10 ng/mL FGF4 + 200 µg/mL AA-treated CMs were assessed by monitoring the light intensity of the selected regions (yellow circles) of interest over a 10 s period. Scale bars = 100 μm. (**C**) Comparison of peak-to-peak durations. *** *p* < 0.001 (n = 63 for control and 80 for FGF4+AA; two-tailed unpaired Student’s *t*-test).

In FGF4+AA-treated group, beating CMs with thick clump-like structures were observed similar to those of FGF4-treated group (Figure 2). Beating properties of FGF4+AA-treated CMs were investigated by analyzing movies taken under a microscope on day 15 of differentiation. Representative beating patterns are shown by the real-time images of beating control CMs and FGF4+AA-treated CMs (Figure 2A,B). Interestingly, untreated CMs showed a heterogeneous beating rate, whereas FGF4+AA-treated CMs showed a more synchronous beating rate (Figure 2A,B; Videos S6 and S7). Peak-to-peak duration of FGF4+AA-treated CMs was significantly homogenous relative to that of the control group (Figure 2C). This result indicated that FGF4+AA co-treatment synchronized contraction of BG01 hESC-CMs, which induced functional maturation of CMs.

### 3.3. AMI Biomarkers Are Released into the Culture Medium of FGF4+AA-Treated BG01 hESC-CMs in Response to Hypoxic Injury 

**Figure 3:**
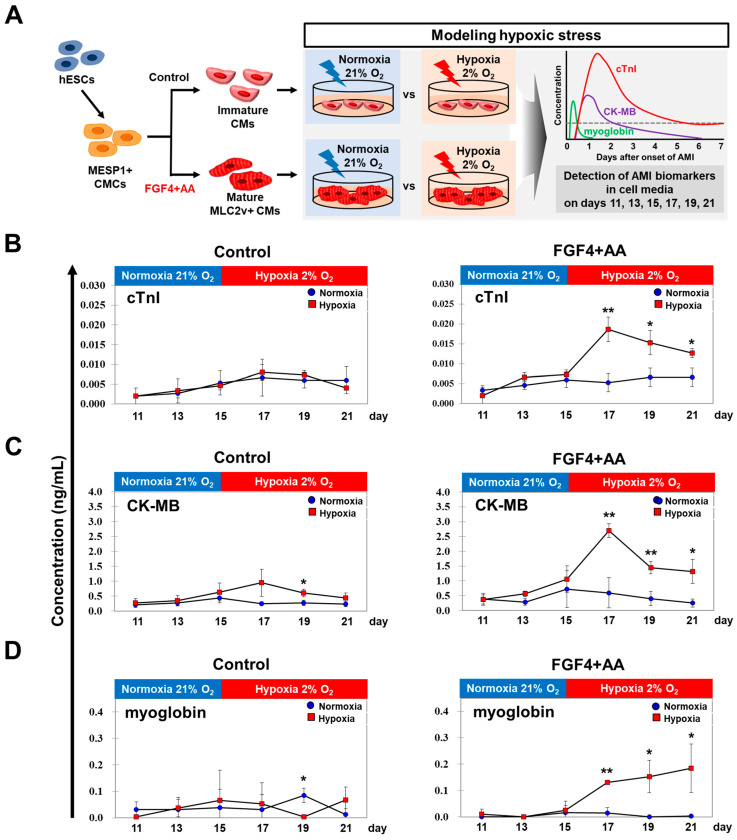
Non-invasive and continuous detection of AMI biomarkers from the culture medium of BG01 hESC-CMs. (**A**) Schematic diagram of detection of hypoxia-induced AMI biomarkers using hESCs cultured without or with 10 ng/mL FGF4 + 200 µg/mL AA between days 5 and 15 of differentiation. Culture media from hESC-CMs grown under normoxic or hypoxic conditions for 24 h without 10 ng/mL FGF4 + 200 µg/mL AA were collected every 2 days between days 11 and 21 of differentiation. CM: cardiomyocyte; CMC: cardiogenic mesoderm cell. AMI specific biomarkers, cTnI (**B**), CK-MB (**C**), and myoglobin (**D**) were evaluated using PATHFAST. Values represent means ± SDs. * *p* < 0.05 and ** *p* < 0.01 versus normoxia. Two-tailed unpaired Student’s *t*-test.

To investigate the feasibility of FGF4+AA-treated BG01 hESC-CMs as an in vitro hypoxic stress model, we evaluated the release of AMI-specific biomarkers (cTnI, CK-MB, and myoglobin) by collecting media samples from normoxia (21% O_2_) or hypoxia (2% O_2_)-treated hESC-CMs cultured without or with FGF4+AA. Culture media from hESC-CMs exposed to normoxia or hypoxia and cultured for 24 h without or with FGF4+AA were collected on days 11, 13, 15, 17, 19, and 21 of differentiation. AMI specific biomarkers (cTnI, CK-MB, and myoglobin) were evaluated using PATHFAST (Figure 3A).

cTnI, CK-MB, and myoglobin levels were very low in samples collected on days 11, 13, and 15 before hypoxic injury in both untreated and FGF4+AA-treated CMs (Figure 3B–D; Figure S5). Hypoxic treatment triggered a strong increase in cTnI, CK-MB, and myoglobin expression on days 17, 19, and 21 of differentiation in the FGF4+AA-treated groups (Figure 3B–D; Figure S5). In FGF4+AA-treated groups, cTnI level was sharply increased by 2.71-fold 2 days after hypoxia, and elevated values were maintained at 2.14-fold at 4 days and 1.86-fold at 6 days after hypoxia compared to the control cells (Figure 3B; Figure S5A). No significant difference in cTnI levels between normoxia and hypoxia in untreated hESC-CMs were found (Figure 3B; Figure S5A). CK-MB levels in FGF4+AA-treated hESC-CMs were increased by approximately 2.55-fold at 2 days after hypoxia, but returned to approximately 1.37- and 1.25-fold of control cell levels at day 4 and day 6 after hypoxia (Figure 3C; Figure S5B). CK-MB levels were slightly increased at 2 and 4 days after hypoxia in untreated hESC-CMs (Figure 3C; Figure S5B). Myoglobin level was sharply increased by 5.24-fold at 2 days after hypoxia, and remained elevated at 6.12-fold and 7.4-fold at 4 and 6 days after hypoxia compared to levels in normoxic FGF4+AA-treated hESC-CMs (Figure 3D; Figure S5C). A slight increase in myoglobin was observed 4 days after hypoxic injury in untreated hESC-CMs (Figure 3D; Figure S5C). These results showed that only FGF4+AA-treated BG01 hESC-CMs robustly released AMI biomarkers into culture medium in response to hypoxic injury, indicating that these cells are appropriate for use as in vitro hypoxic stress model.

### 3.4. Levels of AMI Biomarkers Released into Culture Medium Are Correlated with Sequential Changes in the Contractile Properties of Hypoxia-Exposed BG01 hESC-CMs

We further investigated the relationships between biomarkers (cTnI, CK-MB, and myoglobin) and contractile properties (beat-rate and peak-to-peak duration) of BG01 hESC-CMs during hypoxic injury. Interestingly, we found an apparent correlation between changes in contractile properties and AMI biomarkers (cTnI and CK-MB) during hypoxic injury (Figures S6 and S7). A strong decrease in beat-rate was observed at 2 days after exposure to hypoxia, and these effects were accompanied by a marked increase in cTnI and CK-MB release (Figures S6 and S7). A gradual reduction in cTnI and CK-MB release from days 4 to 6 after hypoxia was positively correlated with changes in contractile properties (Figures S6 and S7). In addition, peak-to-peak duration of FGF4+AA-treated CMs showed an inverse correlation with cTnI and CK-MB release during hypoxia injury (Figures S6 and S7). Myoglobin level was elevated at day 2 in response to hypoxia, whereas higher levels were sustained overall during hypoxia treatment (Figures S6 and S7). The correlation between contractile properties and myoglobin release was less obvious than that between cTnI and CK-MB and hypoxic conditions (Figures S6 and S7).

Next, we evaluated changes in contractile properties (beat-rate and peak-to-peak duration) of hESC-CMs during hypoxic injury. Beat-rate of FGF4+AA-treated hESC-CMs was rapidly reduced during the initial 2 days of acute hypoxia, but gradually recovered over the next 17 days, and returned to normal levels at 25 day (Figure S8A,B). Peak-to-peak duration of FGF4+AA-treated CMs showed a negative correlation with beat-rate (Figure S8A,C).

### 3.5. RNA-Seq Analysis Demonstrated That FGF4+AA-Treated BG01 hESC-CMs Are a Suitable In Vitro Hypoxic Stress Model

**Figure 4:**
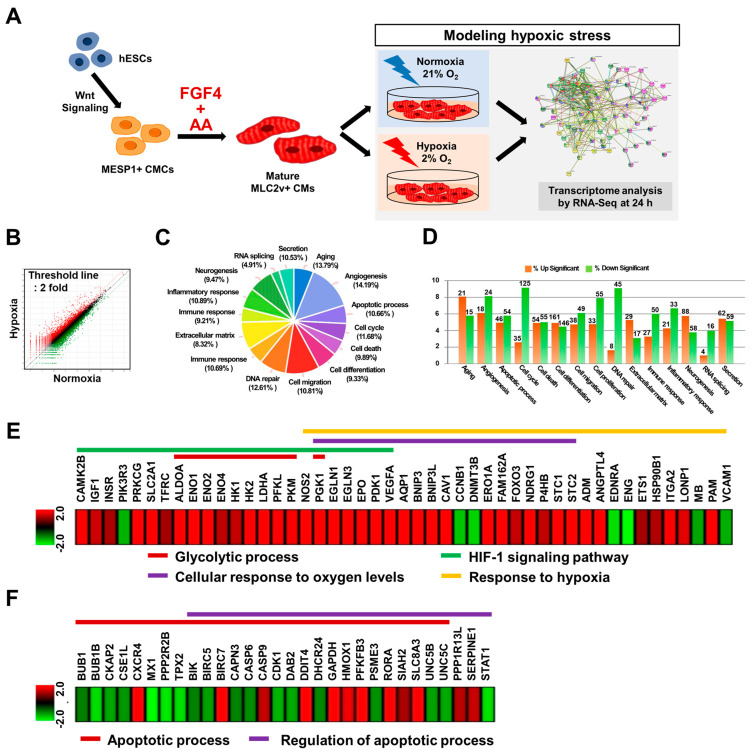
Transcriptome of FGF4+AA-treated BG01 hESC-CMs showing expression of hypoxia-responsive genes. (**A**) Schematic diagram of transcriptome analysis of FGF4+AA-treated hESC-CMs cultured under normoxic (21% O_2_) or hypoxic (2% O_2_) conditions for 24 h. CM: cardiomyocyte; CMC: cardiogenic mesoderm cell. (**B**) Scatter plot showing transcript expression in BG01 hESC-CMs cultured under normoxic- or hypoxic conditions for 24 h after treatment with FGF4+AA between days 5 and 15 of differentiation. (**C**,**D**) Gene ontology analysis of differentially expressed genes in hESC-CMs cultured under hypoxia for 24 h. Clustered heatmaps of differentially expressed genes related to (**E**) glycolytic process, HIF-signaling pathway, cellular response to oxygen levels, and response to hypoxia, and (**F**) apoptotic process, and regulation of apoptotic process in BG01 hESC-CMs cultured under hypoxia for 24 h (fold change 2; log2 normalized read counts of 4 were selected).

To investigate whether FGF4+AA-treated BG01 hESC-CMs are suitable an in vitro hypoxic stress model at the molecular level, we analyzed the transcriptomes of FGF4+AA-treated hESC-CMs cultured under normoxic (21% O_2_) or hypoxic (2% O_2_) conditions for 24 h (Figure 4A). In total, of 25,737 genes, 4673 differentially expressed genes (2731 upregulated and 1942 downregulated genes) with fold changes greater than 2 were found using the ExDEGA program (Figure 4B–D).

We focused on hypoxia-induced genes mimicking the AMI environment in BG01 hESC-CMs exposed to hypoxia using the DAVID, Medline, and KEGG pathway databases. Differentially expressed genes regulating glycolytic processes, hypoxia-inducible factor 1 (HIF-1) signaling, oxygen levels, and apoptotic processes in hESC-CMs by culturing under hypoxia for 24 h were clustered using Euclidean distance correlation and visualized by a heatmap (Figure 4E–F).

**Figure 5:**
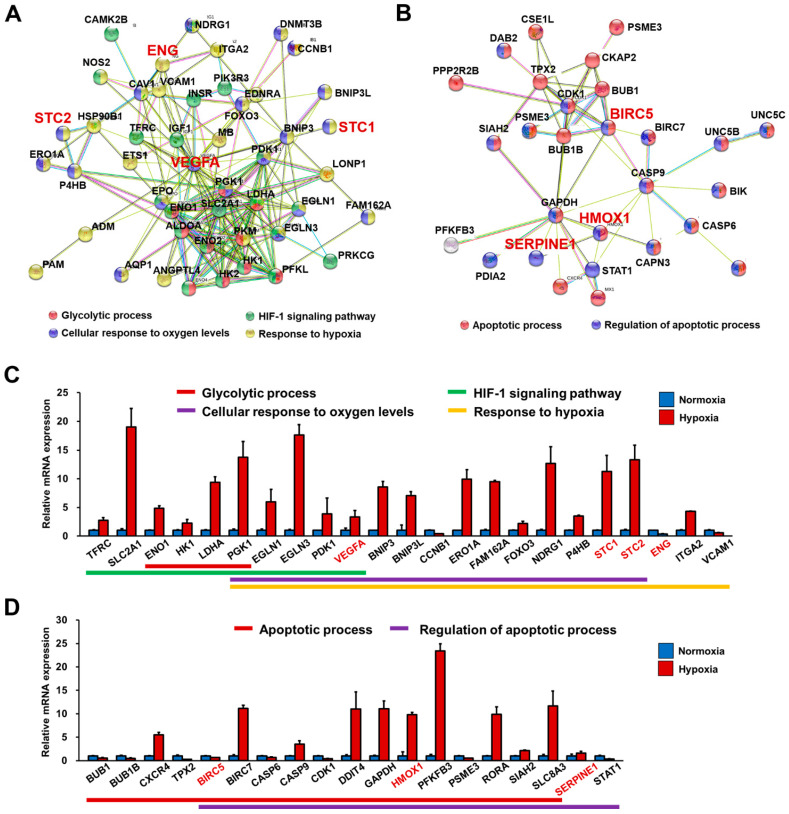
Transcriptome of FGF4+AA-treated BG01 hESC-CMs showing expression of hypoxia-responsive genes. Validation of hypoxia-responsive genes and potential cardiac biomarkers induced in FGF4+AA-treated hESC-CMs by qRT-PCR analysis. Protein–protein interaction networks among differentially expressed genes related to (**A**) glycolytic process, HIF-signaling pathway, cellular response to oxygen levels, and response to hypoxia, and (**B**) apoptotic process, and regulation of apoptotic process in BG01 hESC-CMs. Differentially expressed genes involved in (**C**) hypoxia-responses, and (**D**) apoptotic processes in BG01 hESC-CMs were evaluated using the same samples as those used for RNA-Seq using qRT-PCR. Data were normalized to expression of the housekeeping gene β-ACTIN. Values represent means ± SDs from triplicate data. Potential cardiac biomarkers in the diagnosis and prognosis of coronary artery diseases were indicated with red color in (**A**–**D**).

Genes (ALDOA, ENO1, ENO2, ENO4, HK1, HK2, LDHA, PFKL, PGK1, and PKM) related to glycolysis, which results in the breakdown of glucose and production of pyruvate, were induced in hypoxic hESC-CMs (Figure 4E and Figure 5A; Figure S9). Genes related to cellular responses to oxygen levels (AQP1, BNIP3, BNIP3L, CAV1, CCNB1, DNMT3B, EGLN1, EGLN3, EPO, ERO1A, FAM162A, FOXO3, NDRG1, P4HB, PDK1, PGK1, STC1, STC2, and VEGFA) were induced in hESC-CMs cultured under hypoxia for 24 h (Figure 4E and Figure 5A; Figure S9). In addition, hypoxia-induced genes including HIF-1 signaling pathway genes (CAMK2B, IGF1, INSR, PIK3R3, PRKCG, SLC2A1, and TFRC), and genes related to response to hypoxia (ADM, ATGPTL4, EDNRA, ENG, ETS1, HSP90B1, ITGA2, LONP1, MB, PAM, and VCAM) were expressed by hypoxic hESC-CMs (Figure 4E and Figure 5A; Figure S9).

Results from protein–protein interaction analysis illustrated the genes (BIK, BIRC5, BIRC7, BUB1, BUB1B, CAPN3, CASP6, CASP9, CDK1, CKAP2, CSE1L, CXCR4, DAB2, DDIT4, DHCR24, GAPDH, HMOX1, MX1, PFKFB3, PPP1R13L, PPP2R2B, PSME3, RORA, SERPINE1, SIAH2, SLC8A3, STAT1, TPX2, UNC5B, and UNC5C) involved in apoptotic processes and regulation of apoptotic processes (Figure 4F and Figure 5B; Figure S9). 

We also investigated whether hypoxia-responsive genes identified in our in vitro hypoxic system could function as potential cardiac biomarkers in the diagnosis and prognosis of coronary artery diseases, including unstable angina and AMI. Indeed, we found that BIRC5, ENG, HMOX1, STC1, STC2, SERPINE1, and VEGF induced in response to hypoxia in this study have been reported as potential cardiac biomarkers in clinical studies (Figure 5).

RNA-Seq data were validated by qRT-PCR analysis by evaluating the expression of selected hypoxia-induced genes regulating glycolytic processes, oxygen levels, HIF-1 signaling, and selected apoptotic genes (Figure 5C,D). qRT-PCR analysis showed that the selected genes were upregulated or downregulated in hESC-CMs exposed to hypoxia in a similar manner as to what we observed with RNA-Seq analysis, thus validating the RNA-Seq data (Figure S10). We also found that expression of an apoptotic marker, cleaved caspase 3, was increased and plasma membrane damage was observed in hypoxia-treated hESC-CMs (Figure S11). Taken together, hypoxia treatment of FGF4+AA-treated hESC-CMs modulated the induction of genes associated with hypoxia-induced cellular responses including potential cardiac biomarkers, demonstrating that FGF4+AA-treated BG01 hESC-CMs are a feasible in vitro hypoxic stress model at the molecular level.

## 4. Discussion

Here, we demonstrated for the first time that FGF4 and AA induce differentiation of BG01 hESC-CMCs into mature ventricular CMs at the expense of nodal CMs via screening of soluble factors in a feeder-free culture system. Interestingly, FGF4+AA co-treatment synergistically induced differentiation of BG01 hESC-CMCs into mature ventricular CMs with synchronized contraction. Moreover, FGF4+AA-treated BG01 hESC-CMs robustly released AMI biomarkers (cTnI, CK-MB, and myoglobin) into culture medium in response to hypoxic injury. Hypoxia-responsive genes related to cellular responses to oxygen levels, HIF-1 signaling, glycolytic processes, apoptotic processes, and regulation of cell death were induced in FGF4+AA-treated BG01 hESC-CMs in response to hypoxia based on transcriptome analysis. This study demonstrated the feasibility of in vitro hypoxic stress modeling using hESC-CMs.

In this study, we identified that FGF4 or FGF+AA can induce differentiation of BG01 hESC-CMCs into mature ventricular CMs at the expense of nodal CMs (Figure 1B; Figures S2C and S4B). By contrast, FGF2 markedly induced differentiation into the endothelial cell lineage, whereas FGF10 slightly increased expression of *ANP*, *TBX18*, *HCN4,* and *SM22* in BG01 hESCs (Figure S2C). FGF2 plays roles in self-renewal, survival, and adhesion of hPSCs, and maintains undifferentiated growth of hPSCs in feeder-free conditions in mTesr1 or E8 medium [24,25]. In addition to its proliferative effects on undifferentiated hPSCs, FGF2 could be used to facilitate endothelial differentiation of hPSC-derived CMCs. FGF2 also controls the differentiation of resident cardiac precursors into functional CMs in mice [11]. FGF10 stimulates cardiac differentiation of cultured stem cells [11]. These findings suggest that FGF members modulate lineage specification of hESC-derived CMCs in a FGF member-specific manner.

Although the mechanistic action of FGF4, AA, and FGF4+AA on CM maturation and ventricular CM specification should be determined in future studies, we demonstrated here that FGF4 or AA can induce differentiaton of hESC-CMCs into mature ventricular CMs at the expense of nodal CMs. However, CM maturation and ventricular CM specification in hESC-CMCs treated with FGF4, AA, and FGF4+AA for 10 days was still partial and incomplete compared to adult ventricular CMs. Therefore, longer culture times or a combinatorial approach including soluble factors, extracellular matrix components, and mechanical forces may be required for further maturation and ventricular CM specification.

PATHFAST cTnI-II is a Food and Drug Administration (FDA)-cleared high sensitivity point-of-care troponin assay that has demonstrated similar diagnostic performance for myocardial infarction against a central laboratory high sensitivity assay [16,26,27]. We found correlation coefficients of > 0.98 between the PATHFAST system and an immuno-membrane strip devised to simultaneously measure cTnI, CK-MB, and myoglobin from positive serum [28]. Indeed, we were able to detect AMI biomarkers released into culture medium in response to hypoxic injury using the PATHFAST cardiac biomarker system. 

In this study, we found that genes related to cellular responses to glycolytic processes, oxygen levels, HIF-1 signaling, and apoptotic processes were induced in hESC-CMs cultured under hypoxia for 24 h (Figure 3 and Figure 4; Figure S5). These genes have been previously identified as hypoxia-responsive genes in other cell types including hypoxic CMs as well as animal model of myocardial ischemia and infarction. 

Among hypoxia-responsive genes identified in our in vitro hypoxic system, we also found potential cardiac biomarkers (*BIRC5, ENG, HMOX1, VEGF*, *STC1, STC2,* and *SERPINE1*) which they may have potential clinical value in the diagnosis and prognosis of coronary artery diseases (Figure 4). BIRC5 level was higher in deceased patients, and it increased prediction performance when used together with high sensitivity C-reactive protein (hs-CRP) and heart-type fatty acid binding protein, suggesting that BIRC5 is a potential cardiac biomarker [29,30]. A decrease in ENG level predicted overall cardiovascular mortality in AMI patients [31]. Plasma HMOX1 levels were associated with the severity of coronary heart disease, which was the highest in patients with AMI, followed by unstable angina pectoris and finally stable angina pectoris [32]. Serum VEGF levels on admission in patients with AMI were higher than those in controls and peaked on day 7, and were also higher than in patients with preinfarction angina versus those with no preinfarction angina [33]. STC1 was found to be differentially expressed in culprit coronary plaques of patients with AMI versus those with stable angina [34], and STC2 was shown to be an independent predictor of all-cause death and readmission due to heart failure in ST-segment elevation myocardial infarction [35]. Plasma SERPINE1 levels were elevated in patients with cardiovascular risk factors, and the performance of plasma SERPINE1 was consistent with biomarkers in clinical use (N-terminal pro-B-type natriuretic peptide and CRP) [36]. 

In this study, we developed a FGF4+AA-based protocol to induce differentiation of hESC-CMCs into mature ventricular CMs in a feeder-free culture system. In response to hypoxic injury, AMI biomarkers are robustly released into the culture medium, and hypoxia-response genes and potential AMI biomarkers are identified in mature ventricular hESCs-CMs via transcriptome analyses, demonstrating that FGF4+AA-treated hESC-CMs are a feasible in vitro hypoxic stress model.

## Data Availability

The data that support the findings of this study are available from the corresponding author upon reasonable request.

## Supplementary Materials

The following are available online at https://www.mdpi.com/article/10.3390/cells10102741/s1, Figure S1: Temporal expression patterns of cell lineage markers during cardiogenic mesoderm differentiation of BG01 hESCs, Figure S2: FGF4 and AA enhance gene expression of mature, ventricular, and atrial CM subtype markers, but reduce gene expression of nodal CM and smooth muscle cell markers in BG01 hESC-CMCs, Figure S3: Sequential morphological changes during cardiac differentiation of BG01 hESC-CMCs, Figure S4: Effective FGF4 concentrations at inducing differentiation of BG01 hESC-CMCs into ventricular and atrial CMs, Figure S5: Non-invasive and continuous detection of AMI biomarkers from the culture medium of hESC-CMs, Figure S6: Relationship between AMI biomarkers and contractile properties of BG01 hESC-CMs, Figure S7: Relationship between AMI biomarkers and contractile properties of BG01 hESC-CMs, Figure S8: Sequential changes in the beating properties of BG01 hESC-CMs exposed to hypoxia, Figure S9: Protein-protein interaction networks among differentially expressed genes in hypoxia-induced BG01 hESC-CMs, Figure S10: Validation of differentially expressed genes in hypoxia-induced BG01 hESC-CMs with qRT-PCR, Figure S11: Expression of an apoptotic marker, cleaved caspase 3 (C-cas3), was increased in hypoxia-treated BG01 hESC-CMs, Table S1: Primers used for qRT-PCR, Video S1: BG01 hESC-CMs (control) untreated between days 5 and 15 of differentiation in mCDM3 and RPMI/B27(-Insulin) media, Video S2: BG01 hESC-CMs treated with FGF2 between days 5 and 15 of differentiation in mCDM3 and RPMI/B27(-Insulin) media, Video S3: BG01 hESC-CMs treated with FGF4 between days 5 and 15 of differentiation in mCDM3 and RPMI/B27(-Insulin) media, Video S4: BG01 hESC-CMs treated with FGF10 between days 5 and 15 of differentiation in mCDM3 and RPMI/B27(-Insulin) media, Video S5: BG01 hESC-CMs treated with AA between days 5 and 15 of differentiation in mCDM3 and RPMI/B27(-Insulin) media, Video S6: BG01 hESC-CMs (control) untreated between days 5 and 15 of differentiation in mCDM3 and RPMI/B27(-Insulin) media, Video S7: BG01 hESC-CMs treated with FGF4+AA between days 5 and 15 of differentiation in mCDM3 and RPMI/B27(-Insulin) media.

## References

[1] Yang X., Pabon L., Murry C.E. (2014). Engineering adolescence: Maturation of human pluripotent stem cell-derived cardiomyocytes. Circ. Res..

[2] Lee Y.K., Ng K.M., Chan Y.C., Lai W.H., Au K.W., Ho C.Y., Wong L.Y., Lau C.P., Tse H.F., Siu C.W. (2010). Triiodothyronine promotes cardiac differentiation and maturation of embryonic stem cells via the classical genomic pathway. Mol. Endocrinol..

[3] Chattergoon N.N., Giraud G.D., Louey S., Stork P., Fowden A.L., Thornburg K.L. (2012). Thyroid hormone drives fetal cardiomyocyte maturation. FASEB J..

[4] Yang X., Rodriguez M., Pabon L., Fischer K.A., Reinecke H., Regnier M., Sniadecki N.J., Ruohola-Baker H., Murry C.E. (2014). Tri-iodo-l-thyronine promotes the maturation of human cardiomyocytes-derived from induced pluripotent stem cells. J. Mol. Cell. Cardiol..

[5] Rog-Zielinska E.A., Craig M.A., Manning J.R., Richardson R.V., Gowans G.J., Dunbar D.R., Gharbi K., Kenyon C.J., Holmes M.C., Hardie D.G. (2015). Glucocorticoids promote structural and functional maturation of foetal cardiomyocytes: A role for PGC-1alpha. Cell Death Differ..

[6] Parikh S.S., Blackwell D.J., Gomez-Hurtado N., Frisk M., Wang L., Kim K., Dahl C.P., Fiane A., Tonnessen T., Kryshtal D.O. (2017). Thyroid and Glucocorticoid Hormones Promote Functional T-Tubule Development in Human-Induced Pluripotent Stem Cell-Derived Cardiomyocytes. Circ. Res..

[7] Cao N., Liu Z., Chen Z., Wang J., Chen T., Zhao X., Ma Y., Qin L., Kang J., Wei B. (2012). Ascorbic acid enhances the cardiac differentiation of induced pluripotent stem cells through promoting the proliferation of cardiac progenitor cells. Cell Res..

[8] Karakikes I., Senyei G.D., Hansen J., Kong C.W., Azeloglu E.U., Stillitano F., Lieu D.K., Wang J., Ren L., Hulot J.S. (2014). Small molecule-mediated directed differentiation of human embryonic stem cells toward ventricular cardiomyocytes. Stem Cells Transl. Med..

[9] Iglesias-Garcia O., Baumgartner S., Macri-Pellizzeri L., Rodriguez-Madoz J.R., Abizanda G., Guruceaga E., Albiasu E., Corbacho D., Benavides-Vallve C., Soriano-Navarro M. (2015). Neuregulin-1beta induces mature ventricular cardiac differentiation from induced pluripotent stem cells contributing to cardiac tissue repair. Stem Cells Dev..

[10] Itoh N., Ohta H., Nakayama Y., Konishi M. (2016). Roles of FGF Signals in Heart Development, Health, and Disease. Front. Cell Dev. Biol..

[11] Rosenblatt-Velin N., Lepore M.G., Cartoni C., Beermann F., Pedrazzini T. (2005). FGF-2 controls the differentiation of resident cardiac precursors into functional cardiomyocytes. J. Clin. Investig..

[12] Lopez-Sanchez C., Climent V., Schoenwolf G.C., Alvarez I.S., Garcia-Martinez V. (2002). Induction of cardiogenesis by Hensen’s node and fibroblast growth factors. Cell Tissue Res..

[13] Pradhan A., Zeng X.I., Sidhwani P., Marques S.R., George V., Targoff K.L., Chi N.C., Yelon D. (2017). FGF signaling enforces cardiac chamber identity in the developing ventricle. Development.

[14] Apple F.S., Smith S.W., Pearce L.A., Murakami M.M. (2009). Assessment of the multiple-biomarker approach for diagnosis of myocardial infarction in patients presenting with symptoms suggestive of acute coronary syndrome. Clin. Chem..

[15] Jaffe A.S., Apple F.S. (2012). The third Universal Definition of Myocardial Infarction—Moving forward. Clin. Chem..

[16] Christenson R.H., Mullins K., Duh S.H. (2018). Validation of high-sensitivity performance for a United States Food and Drug Administration cleared cardiac troponin I assay. Clin. Biochem..

[17] Mythili S., Malathi N. (2015). Diagnostic markers of acute myocardial infarction. Biomed. Rep..

[18] Sato Y., Fujiwara H., Takatsu Y. (2012). Cardiac troponin and heart failure in the era of high-sensitivity assays. J. Cardiol..

[19] Lewandrowski K., Chen A., Januzzi J. (2002). Cardiac markers for myocardial infarction. A brief review. Am. J. Clin. Pathol..

[20] Kim D.H., Seo S.M., Cho H.M., Hong S.J., Lim D.S., Paek S.H. (2014). Continuous immunosensing of myoglobin in human serum as potential companion diagnostics technique. Biosens. Bioelectron..

[21] Kim D.H., Paek S.H., Lim G.S., Jeon J.W., Paek S.H. (2012). Performance characteristics of monoclonal antibodies as recyclable binders to cardiac troponin I. Anal. Biochem..

[22] Zhao K., Tang M., Wang H., Zhou Z., Wu Y., Liu S. (2019). Simultaneous detection of three biomarkers related to acute myocardial infarction based on immunosensing biochip. Biosens. Bioelectron..

[23] Howe E.A., Sinha R., Schlauch D., Quackenbush J. (2011). RNA-Seq analysis in MeV. Bioinformatics.

[24] Eiselleova L., Matulka K., Kriz V., Kunova M., Schmidtova Z., Neradil J., Tichy B., Dvorakova D., Pospisilova S., Hampl A. (2009). A complex role for FGF-2 in self-renewal, survival, and adhesion of human embryonic stem cells. Stem Cells.

[25] Chen G., Gulbranson D.R., Yu P., Hou Z., Thomson J.A. (2012). Thermal stability of fibroblast growth factor protein is a determinant factor in regulating self-renewal, differentiation, and reprogramming in human pluripotent stem cells. Stem Cells.

[26] Peacock W.F., Diercks D., Birkhahn R., Singer A.J., Hollander J.E., Nowak R., Safdar B., Miller C.D., Peberdy M., Counselman F. (2016). Can a Point-of-Care Troponin I Assay be as Good as a Central Laboratory Assay? A MIDAS Investigation. Ann. Lab. Med..

[27] Sorensen N.A., Neumann J.T., Ojeda F., Giannitsis E., Spanuth E., Blankenberg S., Westermann D., Zeller T. (2019). Diagnostic Evaluation of a High-Sensitivity Troponin I Point-of-Care Assay. Clin. Chem..

[28] Cho J.H., Kim M.H., Mok R.S., Jeon J.W., Lim G.S., Chai C.Y., Paek S.H. (2014). Two-dimensional paper chromatography-based fluorescent immunosensor for detecting acute myocardial infarction markers. J. Chromatogr. B Analyt. Technol. Biomed. Life Sci..

[29] Markovic D., Jevtovic-Stoimenov T., Cosic V., Stosic B., Dinic V., Markovic-Zivkovic B., Jankovic R.J. (2018). Clinical Utility of Survivin (BIRC5), Novel Cardiac Biomarker, as a Prognostic Tool Compared to High-sensitivity C-reactive Protein, Heart-type Fatty Acid Binding Protein and Revised Lee Score in Elderly Patients Scheduled for Major Non-cardiac Surgery: A Prospective Pilot Study. J. Med. Biochem..

[30] Markovic D.Z., Jevtovic-Stoimenov T., Stojanovic M., Vukovic A.Z., Dinic V., Markovic-Zivkovic B.Z., Jankovic R.J. (2019). Cardiac biomarkers improve prediction performance of the combination of American Society of Anesthesiologists physical status classification and Americal College of Surgeons National Surgical Quality Improvement Program calculator for postoperative mortality in elderly patients: A pilot study. Aging Clin. Exp. Res..

[31] Cruz-Gonzalez I., Pabon P., Rodriguez-Barbero A., Martin-Moreiras J., Pericacho M., Sanchez P.L., Ramirez V., Sanchez-Ledesma M., Martin-Herrero F., Jimenez-Candil J. (2008). Identification of serum endoglin as a novel prognostic marker after acute myocardial infarction. J. Cell. Mol. Med..

[32] Chen S.M., Li Y.G., Wang D.M. (2005). Study on changes of heme oxygenase-1 expression in patients with coronary heart disease. Clin. Cardiol..

[33] Soeki T., Tamura Y., Shinohara H., Tanaka H., Bando K., Fukuda N. (2000). Serial changes in serum VEGF and HGF in patients with acute myocardial infarction. Cardiology.

[34] Lee C.W., Hwang I., Park C.S., Lee H., Park D.W., Kang S.J., Lee S.W., Kim Y.H., Park S.W., Park S.J. (2013). Expression of stanniocalcin-1 in culprit coronary plaques of patients with acute myocardial infarction or stable angina. J. Clin. Pathol..

[35] Cediel G., Rueda F., Oxvig C., Oliveras T., Labata C., de Diego O., Ferrer M., Aranda-Nevado M.C., Serra-Gregori J., Nunez J. (2018). Prognostic value of the Stanniocalcin-2/PAPP-A/IGFBP-4 axis in ST-segment elevation myocardial infarction. Cardiovasc. Diabetol..

[36] Jung R.G., Simard T., Di Santo P., Labinaz A., Moreland R., Duchez A.C., Majeed K., Motazedian P., Rochman R., Jung Y. (2019). Performance of plasminogen activator inhibitor-1 as a biomarker in patients undergoing coronary angiography: Analytical and biological considerations. Diab. Vasc. Dis. Res..

