# Supplementary material for: Modeling Hypoxic Stress In Vitro Using Human Embryonic Stem Cells Derived Cardiomyocytes Matured by FGF4 and Ascorbic Acid Treatment
Source: Cells. 2021 Oct 14;10(10):2741. doi: 10.3390/cells10102741 (PMC8534799; doi:10.3390/cells10102741)
Supplement: Supplementary file 1 [file cells-10-02741-s001.zip › cells-1385826-final-supplementary/Final revision_cells-1385826_Supplemental Information.pdf]

Table S1. Primers used for qRT-PCR

Gene	Forward primer (5′–3′)	Reverse primer (5′–3′)	Product (bp)
ANP	GAGCGGACTGGGCTGTAAC	GGAGCCTCTTGCAGTCTGTC	93
BIRC5	TCTGCTTCAAGGAGCTGGA	AAAGTGCTGGTATTACAGGCGTA	88
BIRC7	GCGCACCTTCCTGTCCTA	GGCCCAGAACACAGTCCA	100
BNIP3	TGCTGCTCTCTCATTTGCTG	GACTCCAGTTCTTCATCAAAAGGT	95
BNIP3L	AATGTCGTCCCACCTAGTCG	AGCTCCACCCAGGAACTGT	117
BUB1	AGCCCAGACAGTAACAGACTCC	GCTCCACTCTGTGGCTTGA	96
BUB1B	CAGTCAGACTCTCAGCATCAAGA	CGAGGCAGAAGAACCAGAGA	94
CASP6	GATGCAGCCTCCGTTTACA	CACAGTTTCCCGGTGAGAATA	93
CASP9	AAGCCCAAGCTCTTTTTCATC	ACTCGTCTTCAGGGGAAGTG	88
CCNB1	CATGGTGCACTTTCCTCCTT	AGGTAATGTTGTAGAGTTGGTGTCC	102
CD31	GAGTCCTGCTGACCCTTCTG	TCAGGTTCTTCCCATTTTG	120
CDK1	TGGATCTGAAGAAATACTTGGATTCTA	CAATCCCCTGTAGGATTTGG	96
cTnI	GCAGATGCCATGATGCAG	CACCTCCCGGTTTTCCTT	114
cTnT	GTCGGCAGCTGCTGTTCT	TCCTCTCTCCAGTCCTCCTCT	124
CXCR4	CCTGCCTGGTATTGTCATCC	AGGATGACTGTGGTCTTGAGG	105
DDIT4	CTGGAGAGCTCGGACTGC	TCCAGGTAAGCCGTGTCTTC	77
DDX41	GGGGATGAGTCCATGCTG	GGCAGTCAGTGATCCGATG	82
EGLN1	CGACCTGATACGCCACTGT	GTTCCATTGCCCGGATAAC	93
EGLN3	ATCGACAGGCTGGTCCTCTA	GATAGCAAGCCACCATTGC	85
ENG	AATGCCATCCTTGAAGTCCA	GTGCCATTTTGCTTGGATG	95
ENO1	TCCCAACATCCTGGAGAATAA	ATGCCGATGACCACCTTATC	90
ERO1A	GGATTCTTGTTTGGCCTCCT	CAGGTACAATCATCCAAGTAACCA	128
FAM162A	CGAAGTTCTGCGCTGGTC	GGAAACATCTCTTTCACATAACCTAA	106
FOXO3	CTGTGAATGAATCTGAGCTTGG	TCCCATTAACAGGAGTGACAAG	103
GAPDH	GAGTCCACTGGCGTCTTCAC	TTCACACCCATGACGAACAT	119
HCN4	GCCGACGGCTCCTACTTT	GTAGAGGCGGCAGTAGGTGT	90
HK1	GACCAAGTTTCTCTCTCAGATCG	CCTAGCTGCTGGAGGATAGC	75
HMOX1	TGAACTCCCTGGAGATGACTC	CCTGCAACTCCTCAAAGAGC	99
ITGA2	GCTGATAATTTGGTCAACCTCA	GAACATTCCCATCCGAAGAG	109
LDHA	TCTCTGTAGCAGATTTGGCAGA	AAGACATCATCCTTTATTCCGTAAA	103
MELK	AAACCAGTTCGTTTAAGGCTTTC	GCGGTCACATCTTCCAGACT	104
MESP1	CCGAGTCCTGGATGCTCTC	AGTCTGGGGACGAGACGAG	96
MIXL1	GGTACCCCGACATCCACTT	GCCTGTTCTGGAACCATACCT	87
MLC2a	GGGTGGTGAACAAGGATGAG	GTGTCAGGGCGAACATCTG	93
MLC2v	GCAGGCGGAGAGGTTTTC	AGTTGCCAGTCACGTCAGG	74
NANOG	GATTTGTGGGCCTGAAGAAA	AAGTGGGTTGTTTGCCTTTG	155
NDRG1	GTTTCCTGGCGTCGTCTC	CATGTCCCTGCTGTCACCT	100
P4HB	CCACGGAGGAGTCTGACCT	TCACGATGTCATCAGCCTCT	129
PDK1	TCCGTTCAATTGGTACAAAGC	CCATGTTCTTCTAGGCCTTTCA	114
PFKFB3	AGCTTGTGCCAAAGGTCACT	TCTAAATCTCCCACCCTCACC	104
PGK1	GCTGGTGGGTTTTTGATGA	TTTAGCTCCGCCCAGGAT	96
POU5F1	AGTGAGAGGCAACCTGGAGA	ACACTCGGACCACATCCTTC	110
PSME3	CCGGCTGTTGATTGAGAAAT	TCTGCAACTGTTTCCTCCTG	114
RORA	TCCCTACTGTTCGTTCACCA	CAGGTTTCCAGATGCGATTT	102
SERPINE1	GCAGCAGGACCGACAAGT	TGTCTTATGGCATCCAGTTAAGC	91
SIAH2	GGCAGTCCTGTTTCCCTGT	CACTTGCAGGAAGCACCAG	135
SLC2A1	GCCCATGTATGTGGGTGAA	AGTCCAGGCCGAACACCT	113
SLC8A3	CCGCATGGTGGATATGAGTT	TCACCCAATACTGGCTTTCC	117
SM22	GGCCAAGGCTCTACTGTCTG	CCCTTGTTGGCCATGTCT	74
SMA	ATCCCCGGGACTAAGACG	CAAAGCCGGCCTTACAGAG	113
STAT1	TTGGCACCTAACGTGCTGT	AGTTCGTACCACTGAGACATCCT	96
STC1	GAGGCGGAGCAGAATGACT	GTTGAGGCAACGAACCACTT	84
STC2	CCTACGTGGACCTCGTGAAC	CCCAGTTCTGCTCACACTGA	96
T	GCTGTGACAGGTACCCAACC	CATGCAGGTGAGTTGTCAGAA	106
TBX18	CCTGGAATTCCCAAGCAAG	GAGGAGCCAGACAAAAGGTG	101

TFRC	CTTTGGAGTTATTAAAGGCTTTGTAGA	CTGTGCCTACACCGGATTTT	104
TPX2	CCATTCCGTCAAATGCTTG	TCCTTCTGAGCAGAAAGCCTA	106
VCAM1	TGCACAGTGACTTGTGGACAT	CCACTCATCTCGATTTCTGGA	92
VEGFA	TGCCCGCTGCTGTCTAAT	TCTCCGCTCTGAGCAAGG	70
VEGFR2	ATGACATTTTGATCATGGAGC	CCCAGATGCCGTGCATGAG	193
β-ACTIN	CTTCTACAATGAGCTGCGTGTGGCTC	GTACATGGCTGGGGTGTTGAAGGTC	108

.

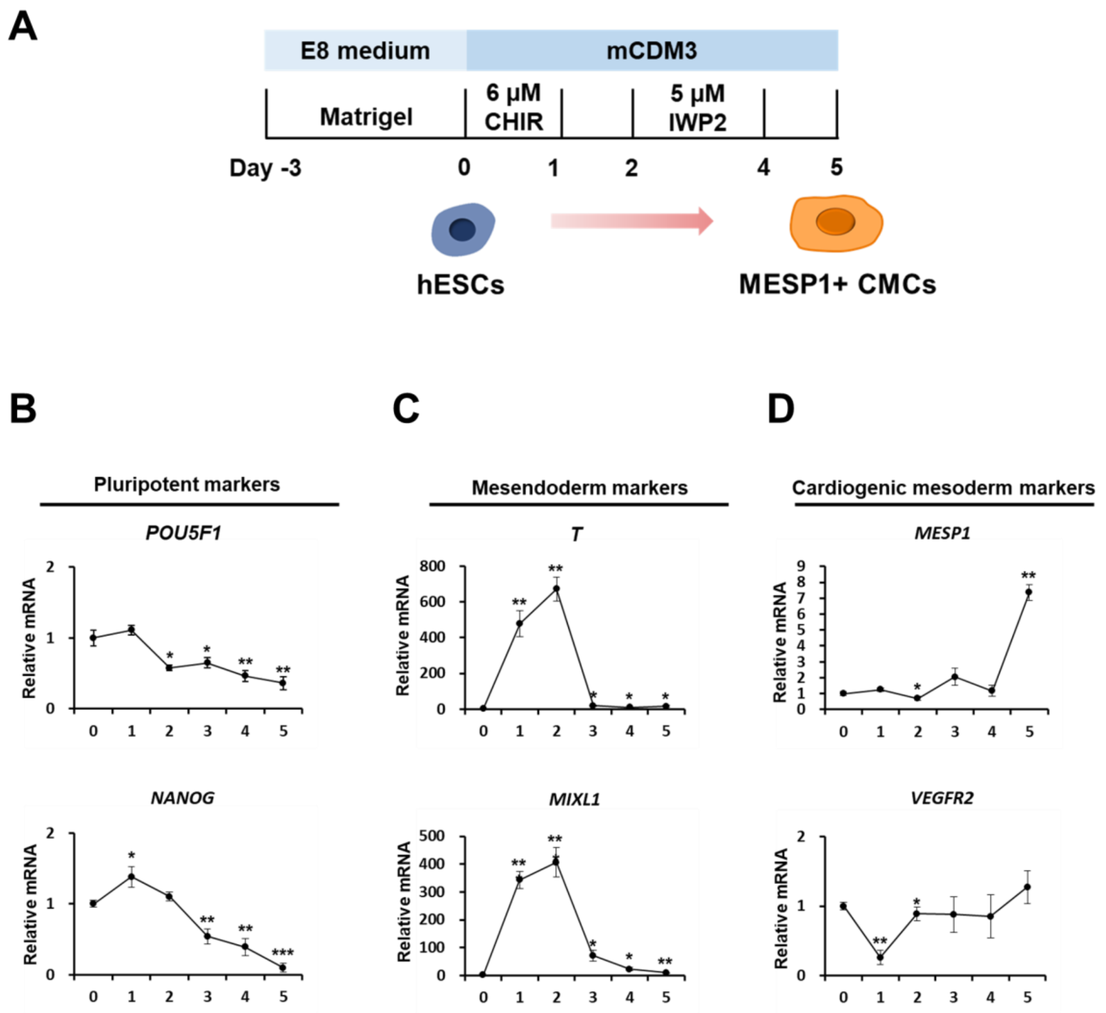

Figure S1. Temporal expression patterns of cell lineage markers during cardiogenic mesoderm differentiation of BG01 hESCs. (**A**) Schematic diagram of cardiogenic mesoderm differentiation protocol for BG01 hESCs along with Wnt modulation in mCDM3 medium. CHIR, CHIR99021; CMC, cardiogenic mesoderm cell; mCDM3, modified chemically defined medium consisting of three components; E8, essential 8. Gene expression in samples undergoing cardiogenic mesoderm differentiation from BG01 hESCs in mCDM3 at the indicated time points was analyzed with a focus on genes associated with (**B**) pluripotency (*POU5F1*, *NANOG*), (**C**) mesendoderm (*T*, *MIXL1*), and (**D**) cardiogenic mesoderm (*MESP1*, *VEGFR2*). Data were normalized to *GAPDH* level and expressed as relative values. Three independent experiments were performed. Values represent means ± SDs. $n = 3$. *$p < 0.05$, **$p < 0.01$ and ***$p < 0.001$ versus day 1. Two-tailed unpaired Student's t-test.

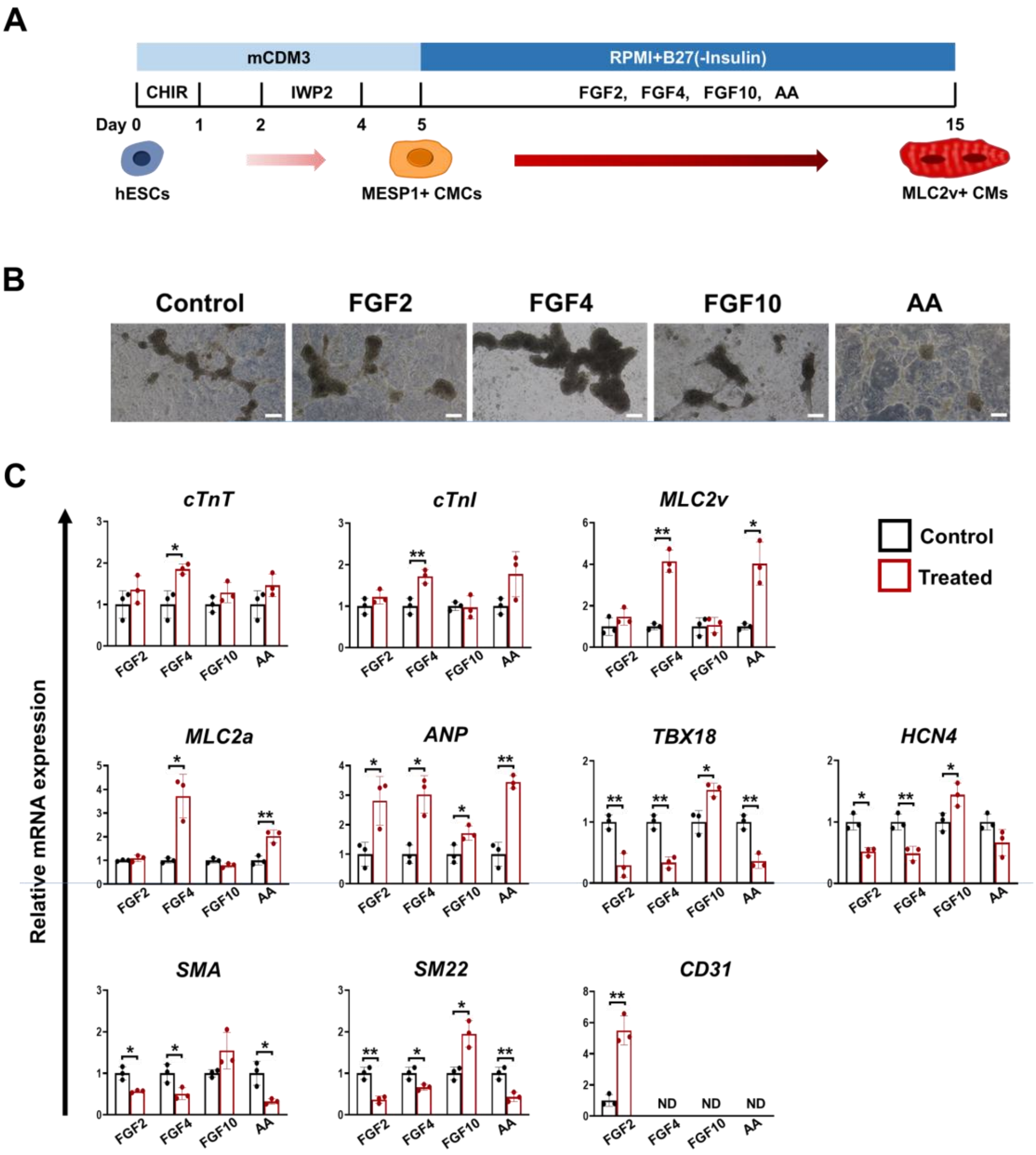

Figure S2. FGF4 and AA enhance gene expression of mature, ventricular, and atrial CM subtype markers, but reduce gene expression of nodal CM and smooth muscle cell markers in BG01 hESC-CMCs. (**A**) Schematic diagram of CM subtype differentiation protocol from BG01 hESC along with Wnt modulation followed by 10 ng/mL FGF2, 10 ng/mL FGF4, 10 ng/mL FGF10, or 200 μg/mL AA between days 5 and 15 of differentiation in mCDM3 and RPMI/B27(-Insulin) media. CHIR: CHIR99021; CM: cardiomyocyte; CMC: cardiogenic mesoderm cell; mCDM3: modified chemically defined medium consisting of three components. (**B**) Morphology of hESC-CMs treated with 10 ng/mL FGF2, 10 ng/mL FGF4, 10 ng/mL FGF10, or 200 μg/mL AA. Scale bars = 200 μm. (**C**) qRT-PCR analysis of a total CM marker (cTnT), a mature CM marker (cTnI), a ventricular CM marker (MLC2v), atrial CM markers (MLC2a, ANP), nodal CM markers (TBX18, HCN4), smooth muscle cell markers (SMA, SM22), and an endothelial cell marker (CD31) in BG01 hESC-CMs treated with 10 ng/mL FGF2, 10 ng/mL FGF4, 10 ng/mL FGF10, or 200 μg/mL AA in mCDM3 and RPMI/B27(-Insulin) media at differentiation day 15. Data were normalized to GAPDH level and expressed as relative values. Values represent means ± SDs. $n = 3$. ND: not detected. *$p < 0.05$ and **$p < 0.01$ versus controls. Two-tailed unpaired Student's t-test.

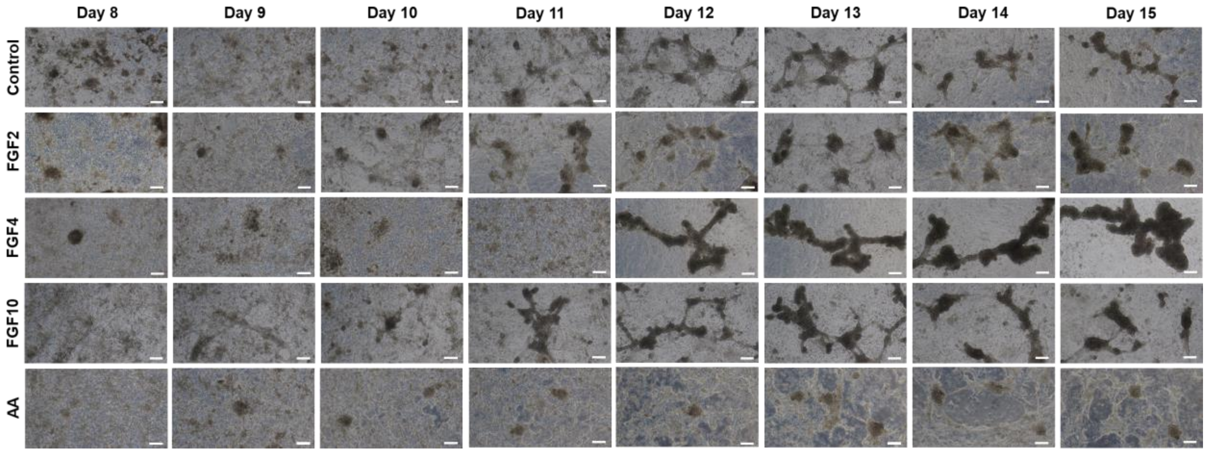

Figure S3. Sequential morphological changes during cardiac differentiation of BG01 hESC-CMCs. Morphological changes during hESC-CMC differentiation toward a cardiac lineage after treatment with FGF2, FGF4, FGF10, or AA in mCDM3 and RPMI/B27(-Insulin) media from days 8 to 15. Scale bar, 200 μm.

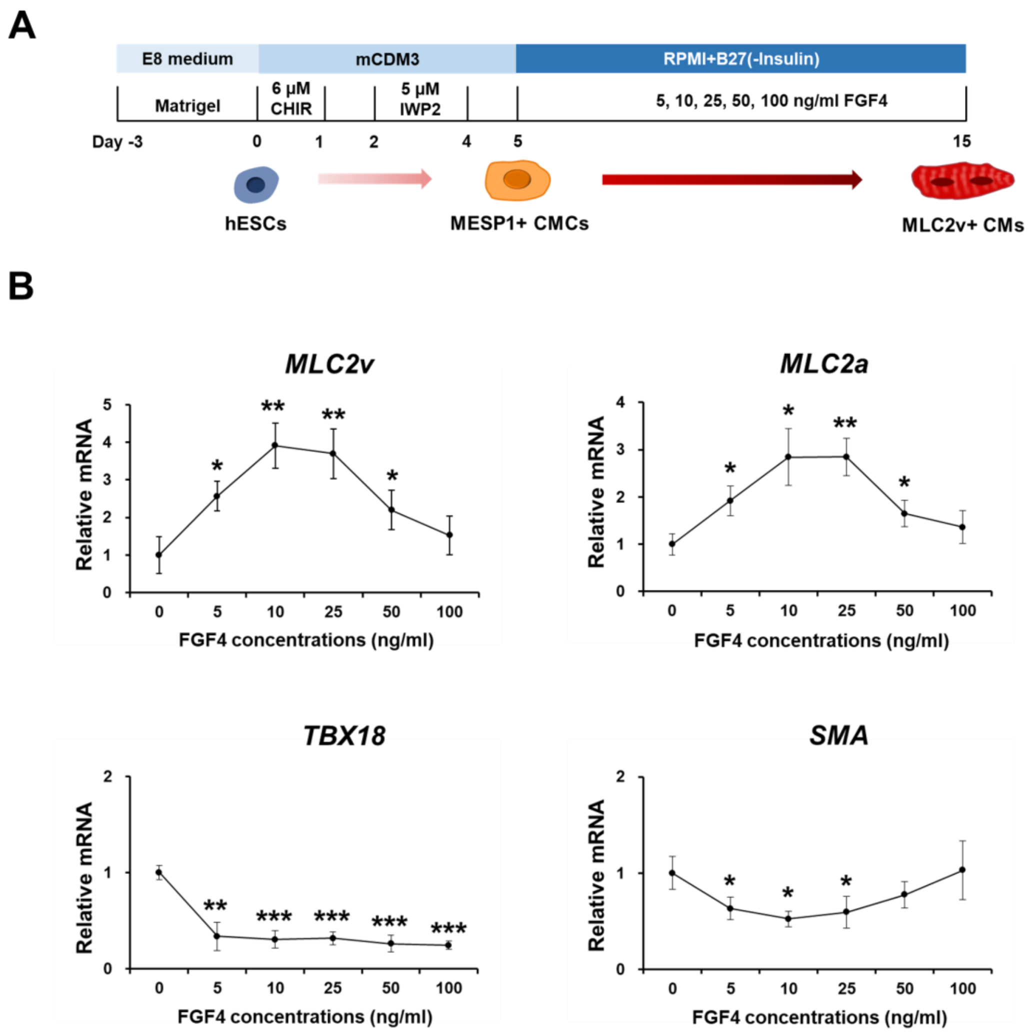

Figure S4. Effective FGF4 concentrations at inducing differentiation of BG01 hESC-CMCs into ventricular and atrial CMs. (**A**) Schematic diagram of the protocol used for CM subtype differentiation from hESC-CMCs treated with different concentrations of FGF4 in mCDM3 and RPMI/B27(-Insulin) media. CHIR, CHIR99021; CMC, cardiogenic mesoderm cell; mCDM3, modified chemically defined medium consisting of three components; E8, essential 8. (**B**) qRT-PCR analysis for a ventricular CM marker (*MLC2v*), an atrial CM marker (*MLC2a*), a nodal CM marker (*TBX18*), and a smooth muscle cell marker (*SMA*) in BG01 hESC-CMCs treated with different concentrations of FGF4 in mCDM3 and RPMI/B27(-Insulin) media at differentiation day 15. Data were normalized to *GAPDH* level and expressed as relative values. Values represent means ± SDs. $n = 3$. *$p < 0.05$, **$p < 0.01$ and ***$p < 0.001$ versus controls. Two-tailed unpaired Student's *t*-test.

A

cTnI

Control	AVE		SD	
	Normoxia	Hypoxia	Normoxia	Hypoxia
Day 11	0.002	0.002	0.002	0.000
Day 13	0.003	0.003	0.001	0.003
Day 15	0.005	0.005	0.003	0.001
Day 17	0.007	0.008	0.005	0.002
Day 19	0.006	0.007	0.002	0.001
Day 21	0.006	0.004	0.003	0.000

FGF4+AA	AVE		SD	
	Normoxia	Hypoxia	Normoxia	Hypoxia
Day 11	0.003	0.002	0.001	0.002
Day 13	0.005	0.007	0.001	0.001
Day 15	0.006	0.007	0.002	0.001
Day 17	0.005	0.019	0.002	0.003
Day 19	0.007	0.015	0.002	0.003
Day 21	0.007	0.013	0.002	0.001

B

CK-MB

Control	AVE		SD	
	Normoxia	Hypoxia	Normoxia	Hypoxia
Day 11	0.208	0.265	0.042	0.151
Day 13	0.269	0.347	0.082	0.185
Day 15	0.433	0.636	0.159	0.307
Day 17	0.249	0.944	0.054	0.451
Day 19	0.267	0.603	0.089	0.129
Day 21	0.226	0.441	0.084	0.162

FGF4+AA	AVE		SD	
	Normoxia	Hypoxia	Normoxia	Hypoxia
Day 11	0.383	0.378	0.197	0.155
Day 13	0.289	0.564	0.111	0.099
Day 15	0.721	1.055	0.623	0.452
Day 17	0.595	2.693	0.511	0.231
Day 19	0.400	1.448	0.238	0.203
Day 21	0.251	1.322	0.140	0.412

C

myoglobin

Control	AVE		SD	
	Normoxia	Hypoxia	Normoxia	Hypoxia
Day 11	0.031	0.003	0.029	0.006
Day 13	0.031	0.037	0.046	0.033
Day 15	0.039	0.066	0.068	0.114
Day 17	0.032	0.053	0.055	0.079
Day 19	0.085	0.004	0.027	0.007
Day 21	0.013	0.068	0.022	0.048

FGF4+AA	AVE		SD	
	Normoxia	Hypoxia	Normoxia	Hypoxia
Day 11	0.000	0.011	0.000	0.018
Day 13	0.000	0.000	0.000	0.000
Day 15	0.016	0.025	0.028	0.034
Day 17	0.015	0.131	0.020	0.004
Day 19	0.000	0.153	0.000	0.061
Day 21	0.003	0.185	0.005	0.091

Figure S5. Non-invasive and continuous detection of AMI biomarkers from the culture medium of BG01 hESC-CMs. Culture media from normoxia- or hypoxia-treated BG01 hESC-CMs cultured for 24 h with or without FGF4+AA were collected on days 11, 13, 15, 17, 19, and 21 of differentiation. AMI specific biomarkers (cTnI, CK-MB, and myoglobin) were evaluated using PATHFAST.

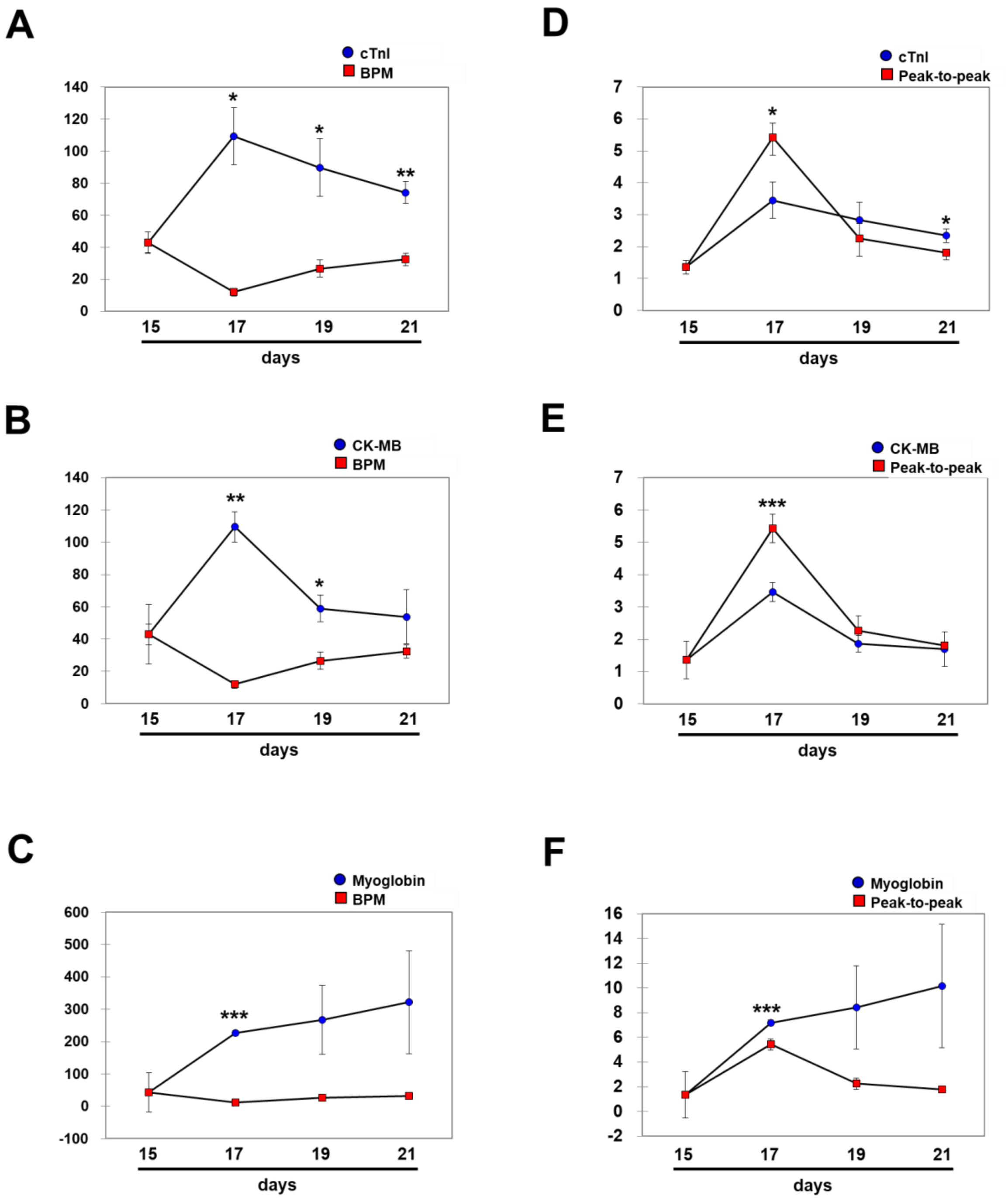

Figure S6. Relationship between AMI biomarkers and contractile properties of BG01 hESC-CMs. Relationships between normalized cTnI (**A**, **D**), CK-MB (**B**, **E**), myoglobin (**C**, **F**) release, and contractile properties (beat-rate and peak-to-peak duration) of BG01 hESC-CMs during hypoxic injury are shown. Values represent means ± SDs. *$p < 0.05$ and **$p < 0.01$ versus controls. Two-tailed unpaired Student's t-test.

A

Normalized cTnI (fold-change)

BPM

	AVE		SD	
	Normalized cTnI	BPM	Normalized cTnI	BPM
Day 15	42.932	42.932	6.760	6.463
Day 17	109.281	11.957	17.885	2.407
Day 19	89.767	26.560	17.885	5.387
Day 21	74.155	32.331	6.760	3.969

Peak-to-peak

	AVE		SD	
	Normalized cTnI	Peak-to-peak	Normalized cTnI	Peak-to-peak
Day 15	1.357	1.357	0.214	0.054
Day 17	3.453	5.430	0.565	0.446
Day 19	2.837	2.264	0.565	0.457
Day 21	2.343	1.809	0.214	0.113

B

Normalized CK-MB (fold-change)

BPM

	AVE		SD	
	Normalized CK-MB	BPM	Normalized CK-MB	BPM
Day 15	42.931	42.932	18.399	6.463
Day 17	109.565	11.957	9.395	2.407
Day 19	58.905	26.560	8.277	5.387
Day 21	53.779	32.331	16.750	3.969

Peak-to-peak

	AVE		SD	
	Normalized CK-MB	Peak-to-peak	Normalized CK-MB	Peak-to-peak
Day 15	1.357	1.357	0.582	0.054
Day 17	3.464	5.430	0.297	0.446
Day 19	1.862	2.264	0.262	0.457
Day 21	1.700	1.809	0.530	0.113

C

Normalized myoglobin (fold-change)

BPM

	AVE		SD	
	Normalized myoglobin	BPM	Normalized myoglobin	BPM
Day 15	42.932	42.932	59.922	6.463
Day 17	227.423	11.957	7.246	2.407
Day 19	266.874	26.560	106.743	5.387
Day 21	321.409	32.331	158.769	3.969

Peak-to-peak

	AVE		SD	
	Normalized myoglobin	Peak-to-peak	Normalized myoglobin	Peak-to-peak
Day 15	1.357	1.357	1.894	0.054
Day 17	7.187	5.430	0.229	0.446
Day 19	8.433	2.264	3.373	0.457
Day 21	10.157	1.809	5.017	0.113

Figure S7. Relationship between AMI biomarkers and contractile properties of BG01 hESC-CMs. Relationships between normalized cTnI (**A**), CK-MB (**B**), myoglobin (**C**) release, and contractile properties (beat-rate and peak-to-peak duration) of BG01 hESC-CMs during hypoxic injury are shown.

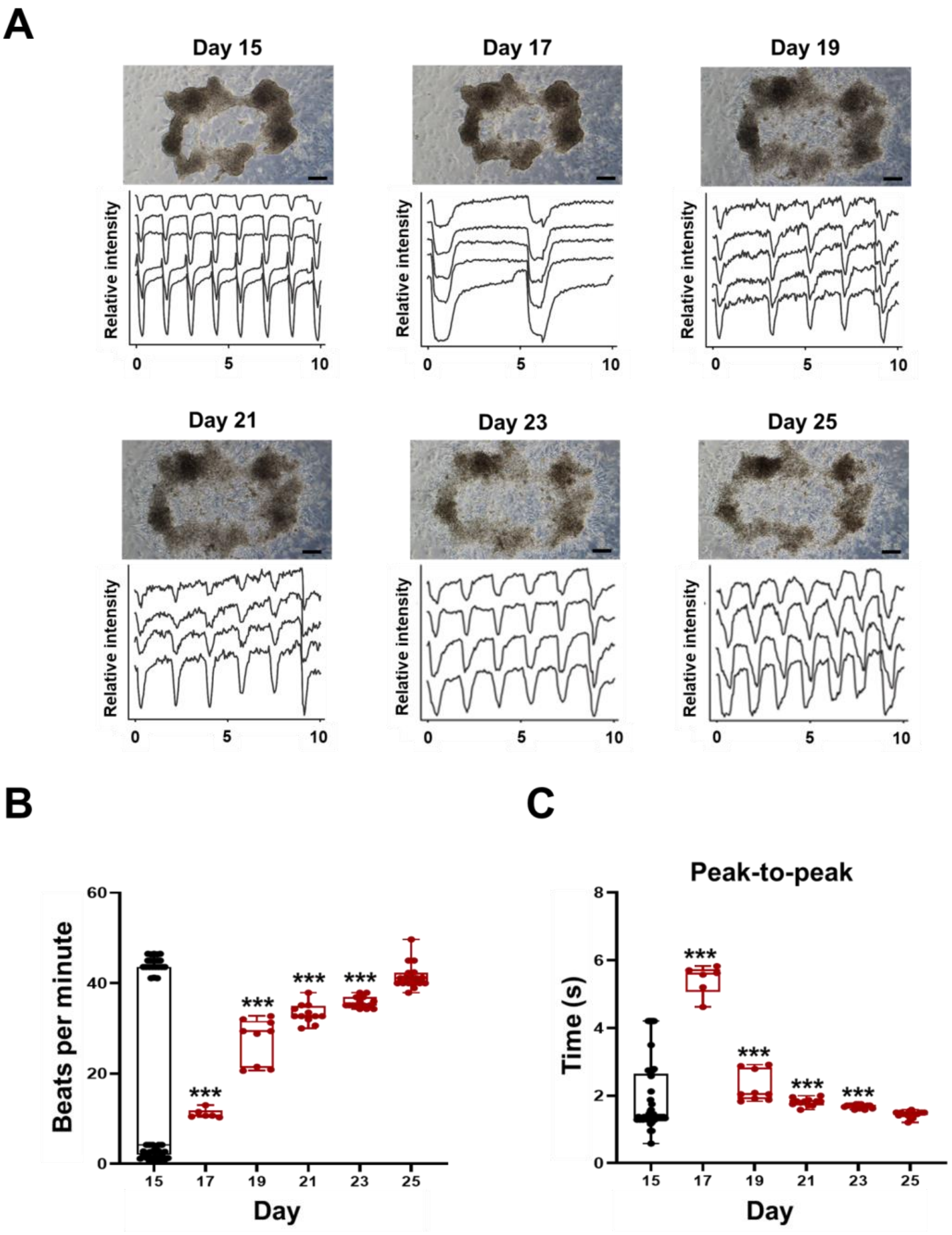

Figure S8. Sequential changes in the beating properties of BG01 hESC-CMs exposed to hypoxia. Temporal changes in morphology (**A**), beats per minutes (**B**), and peak-to-peak (**C**) of FGF4+AA-treated hESC-CMs cultured under hypoxia (2% O_2) were video-recorded every 2 days between days 15 and 25 of differentiation. Scale bars = 200 μm. Values represent means ± SDs. ****p* < 0.001 versus day 15. Significant differences between the means were analyzed by a one-way ANOVA followed by the Student–Newman–Keuls test. *n* = 21, 6, 9, 12, 15, and 18 for beating analysis on days 15, 17, 19, 21, 23, and 25, respectively.

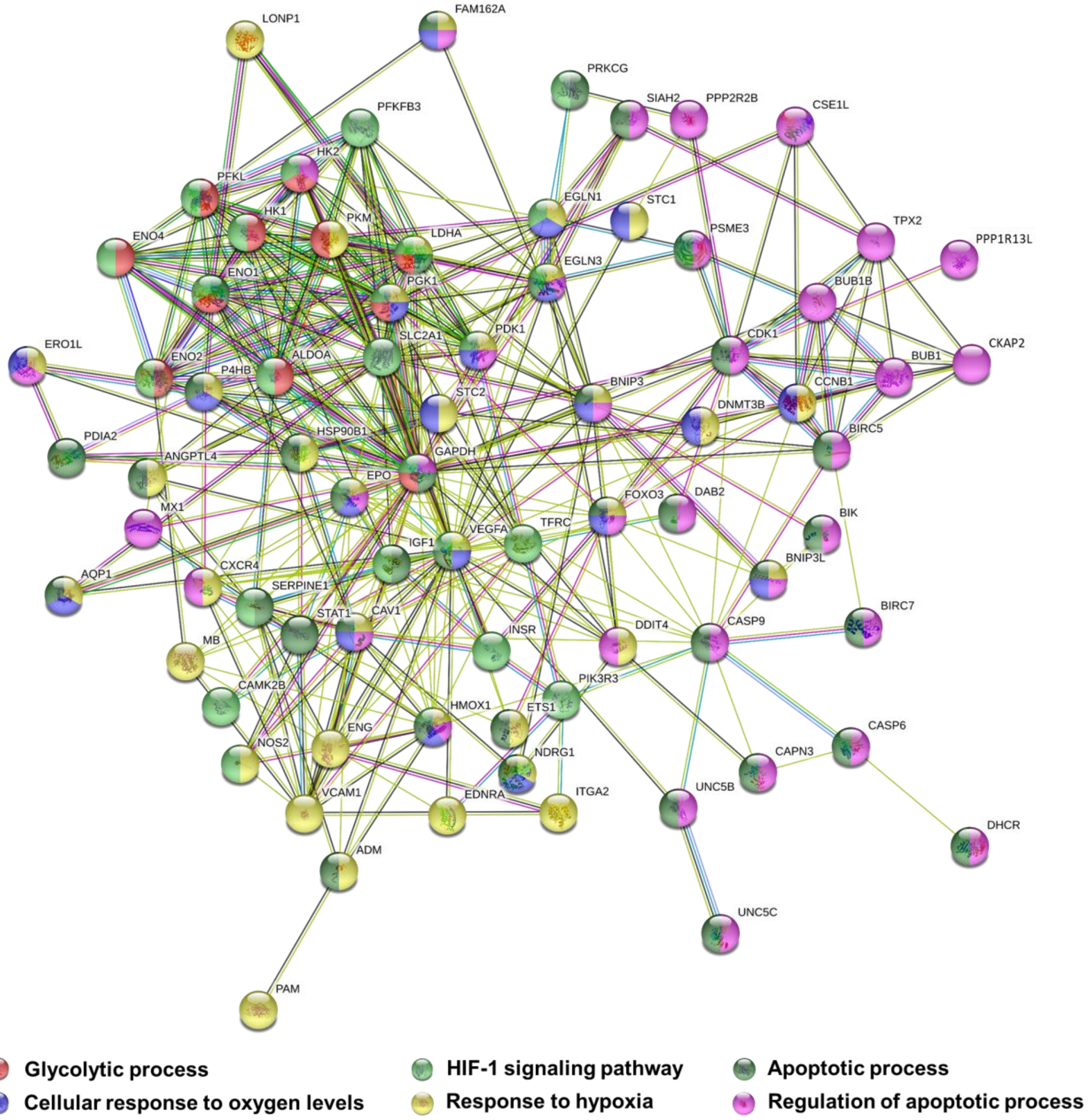

Figure S9. Protein-protein interaction networks among differentially expressed genes in hypoxia-induced BG01 hESC-CMs. The STRING search tool was used to generate protein-protein interaction networks in differentially expressed genes related to glycolytic process, cellular response to oxygen levels, and HIF-1 signaling pathway, response to hypoxia, apoptotic process, and regulation of apoptotic process in BG01 hESC-CMs cultured under hypoxia for 24 h.

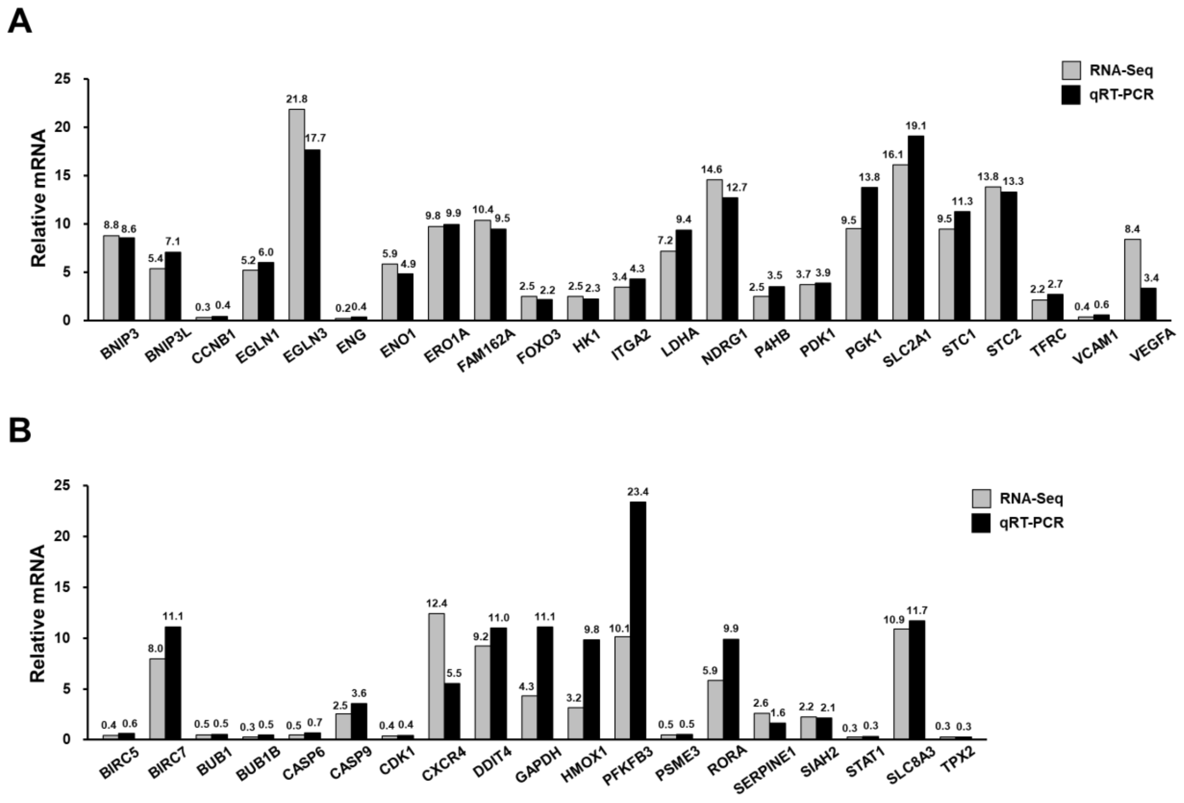

Figure S10. Validation of differentially expressed genes in hypoxia-induced BG01 hESC-CMs with qRT-PCR. Relative expression of genes involved in (**A**) responses to hypoxia including glycolytic process, cellular response oxygen levels, and HIF-1 signaling pathway, and (**B**) apoptotic processes was analyzed by qRT-PCR using the same samples as used for RNA-Seq. Data were normalized to *β-ACTIN* level and expressed as relative values. Values represent means ± SDs from triplicate data.

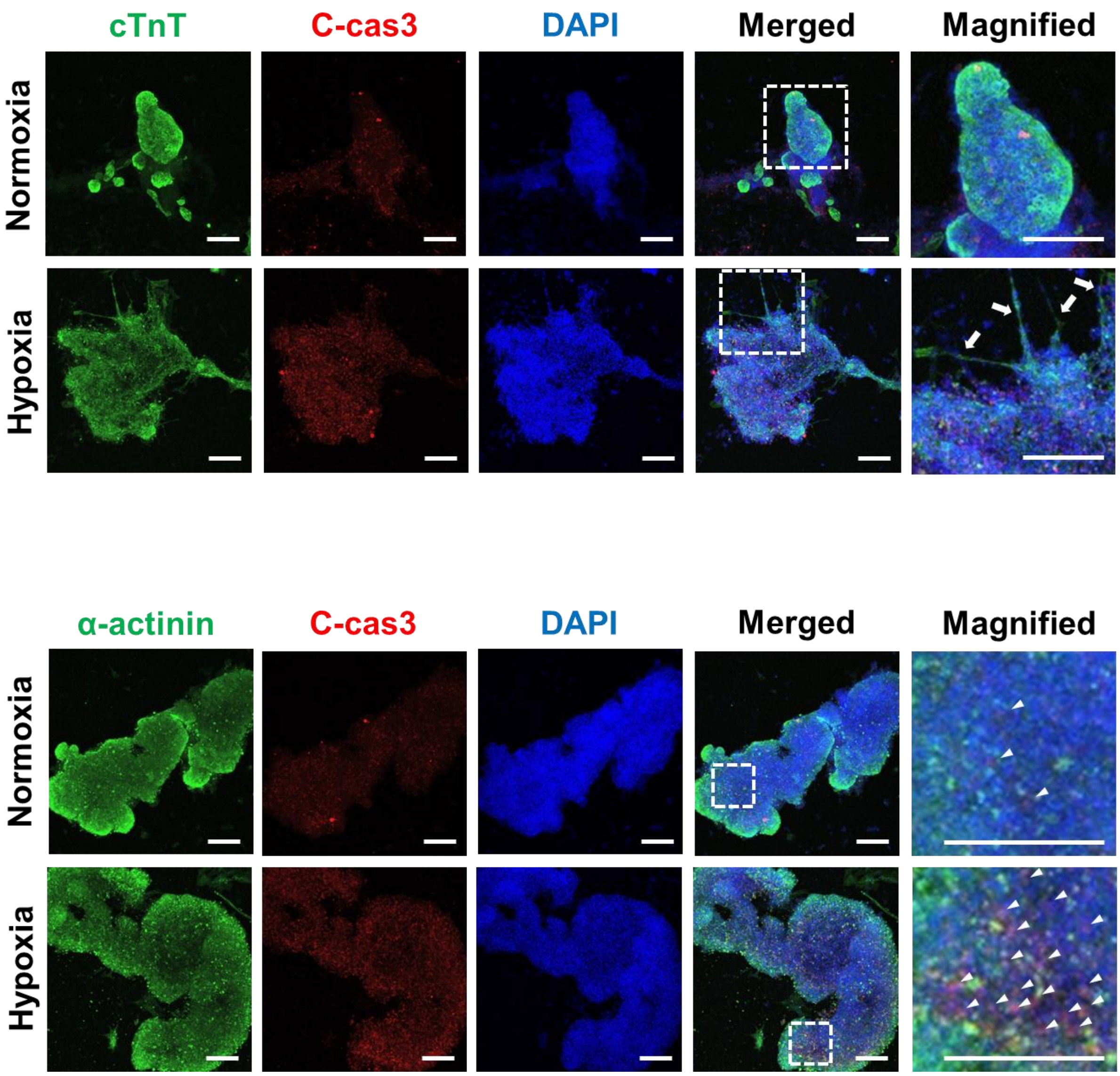

Figure S11. Expression of an apoptotic marker, cleaved caspase 3 (C-cas3), was increased in hypoxia-treated BG01 hESC-CMs. Immunofluorescence images of hESC-CMs treated with FGF4+AA at day 15. Primary antibody staining followed by cTnT (green) or α-actinin (green) and C-cas3 (red). Nuclei were stained with DAPI (blue). White arrows indicate damage of cell membranes. White arrowheads indicate C-cas3-positive cells. Scale bar, 100 μm.

Legends to Supplemental Videos

Video S1. BG01 hESC-CMs (control) untreated between days 5 and 15 of differentiation in mCDM3 and RPMI/B27(-Insulin) media.

Video S2. BG01 hESC-CMs treated with FGF2 between days 5 and 15 of differentiation in mCDM3 and RPMI/B27(-Insulin) media. .

Video S3. BG01 hESC-CMs treated with FGF4 between days 5 and 15 of differentiation in mCDM3 and RPMI/B27(-Insulin) media.

Video S4. BG01 hESC-CMs treated with FGF10 between days 5 and 15 of differentiation in mCDM3 and RPMI/B27(-Insulin) media.

Video S5. BG01 hESC-CMs treated with AA between days 5 and 15 of differentiation in mCDM3 and RPMI/B27(-Insulin) media.

Video S6. BG01 hESC-CMs (control) untreated between days 5 and 15 of differentiation in mCDM3 and RPMI/B27(-Insulin) media.

Video S7. BG01 hESC-CMs treated with FGF4+AA between days 5 and 15 of differentiation in mCDM3 and RPMI/B27(-Insulin) media.
